# Arctic geese in newly colonised, colder breeding areas have higher spring body mass and breed earlier relative to the onset of spring

**DOI:** 10.1111/1365-2656.70172

**Published:** 2025-11-11

**Authors:** Kees H. T. Schreven, Tom S. L. Versluijs, Michiel P. Boom, Fred Cottaar, Eckhart Kuijken, Jorma Pessa, Ingunn M. Tombre, Christine Verscheure, Jesper Madsen, Bart A. Nolet

**Affiliations:** ^1^ Department of Animal Ecology Netherlands Institute of Ecology (NIOO‐KNAW) Wageningen The Netherlands; ^2^ Department of Theoretical and Computational Ecology Institute of Biodiversity and Ecosystem Dynamics (IBED), University of Amsterdam Amsterdam The Netherlands; ^3^ Department of Coastal Systems Royal Netherlands Institute for Sea, Research (NIOZ), ‘t Horntje Texel The Netherlands; ^4^ Groningen Institute for Evolutionary Life Sciences (GELIFES), University of Groningen Groningen The Netherlands; ^5^ Sovon Dutch Center for Field Ornithology Nijmegen The Netherlands; ^6^ Independent Researcher Haarlem The Netherlands; ^7^ Terrestrial Ecology Unit, Department of Biology Ghent University Ghent Belgium; ^8^ Independent Researcher Beernem Belgium; ^9^ ELY‐Keskus‐Centre for Economic Development, Transport and the Environment Oulu Finland; ^10^ Department for Arctic Ecology The Norwegian Institute for Nature Research (NINA) Tromsø Norway; ^11^ Department of Ecoscience Aarhus University Aarhus Denmark

**Keywords:** breeding success, capital breeder, individual quality, laying date, NDVI, range expansion, snowmelt, vegetation green‐up

## Abstract

1. Global warming causes spring onset to advance, especially in the Arctic. Migratory animals may respond by advancing their phenology or colonising colder areas where spring starts later. The role of climate change in range expansion can be both driving (making traditional areas suboptimal) and facilitating (making new areas suitable).
2. Recently, Pink‐footed Geese (*Anser brachyrhynchus*) from Svalbard showed extreme range expansion by colonising the colder Novaya Zemlya as breeding ground, involving a new migration route. We examine potential costs and benefits associated with breeding in this new area.
3. We use GPS‐tracking, long‐term population monitoring and remote sensing, to compare spring onset, migration timing and breeding performance between both flyways, and to evaluate how spring onset affects different reproductive stages in both breeding areas in these capital breeders.
4. The traditional route showed challenges for migration timing, as spring had advanced in Svalbard (while arrival date had not kept up) but not on stopovers, and recently, early spring on Svalbard correlated with late spring on stopovers. On new stopovers, spring did advance. Spring was later in Novaya Zemlya than Svalbard, yet arrival dates were similar. In Novaya Zemlya, egg laying occurred later than in Svalbard, but still earlier relative to local spring onset (e.g. snowmelt and green‐up), suggesting a smaller mismatch. The period between arrival and egg laying was longer in Novaya Zemlya than Svalbard, but breeding performance was similar. Finally, on the new route, geese were larger and (relatively) heavier than on the traditional route, thus possibly carrying larger capital body stores to cover harsher pre‐laying periods.
5. Our results suggest that colonising new breeding areas enables populations to regain phenological match with the environment, especially when advancement of migration timing was limited. Still, breeding in a colder area may require more parental investment, such as body stores. Thus, a benefit for offspring comes at a cost for parents.
6. This mechanism can cause climate change to drive and facilitate colonisation especially in individuals capable of large investments. Consequently, variation in individual quality leads to heterogeneous effects of climate change within a population. These processes may play at the ‘cold’ edge of any range shift.

## Graphical abstract

Moving to colder areas allows arctic geese to reduce the negative effects of climate change (trophic mismatch), but this comes at a price for parents as they face harsher nesting conditions (snow cover), which only parents capable of large investments can cope with: climate change effects differ between individuals within populations.

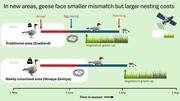

## 1 INTRODUCTION

The Earth's climate is changing, with the fastest change occurring in the Arctic (IPCC, 2021). Rising temperatures lead to changes in the timing of spring events, such as an advancing snowmelt (Stone et al., 2002) and vegetation green‐up (Zhang et al., 2007). In turn, animals can respond in time or in space.

A response in time occurs when seasonal activities of animals advance, for example arthropod emergence (Høye et al., 2007; Visser et al., 1998) or bird spring migration (Jonzén et al., 2006; Rainio et al., 2006; Schmaljohann & Both, 2017) and breeding (Both et al., 2009; Crick et al., 1997). In some migratory birds, advancements did not keep up with the start of spring, resulting in a progressively later arrival and breeding relative to local spring onset (e.g. Both & Visser, 2001; Mayor et al., 2017; Saino et al., 2011; Zhemchuzhnikov et al., 2021). This leads to trophic mismatches if offspring grow up after their food peak and experience lower growth and survival (e.g. Doiron et al., 2015; Lameris et al., 2018; Ross et al., 2018). Constraints for advancement are partly determined by the migratory route. Speeding up migration, by spending less time on stopovers, is an important way for arctic birds to adjust to a warming Arctic (Lameris et al., 2025). However, when stopover and breeding area are far apart or separated by an ecological barrier (e.g. a sea), they are subject to different rates of climate change (Clausen & Clausen, 2013). This limits possibilities to advance fuelling and to forecast spring onset in the breeding area from the stopover (Kölzsch et al., 2015).

A response in space occurs when animals colonise new, colder breeding areas, where springs starts later and better matches their phenology (e.g. Hušek et al., 2014; conceptual: Lamers et al., 2023). Such range shifts could occur towards different habitats (Camacho et al., 2013), north‐facing slopes (Baur & Baur, 2013), higher altitudes (Chen et al., 2011) or higher latitudes (Parmesan & Yohe, 2003). In the Arctic, options for shifting towards colder areas are limited and bird populations may show range contraction (Wauchope et al., 2017). Still, in the Eurasian Arctic, spring onset shows a gradient from the Atlantic eastwards (Karlsen et al., 2014), from warm Gulf stream to cold Arctic ocean influence (Polyakov et al., 2017).

Avian breeding range shifts outside the existing annual range are usually slow, gradual processes, over land (Hitch & Leberg, 2007; Lehikoinen & Virkkala, 2016). Breeding range shifts inside the annual range can be faster, such as a redistribution within the existing range (Rakhimberdiev et al., 2011) or breeding inside the wintering range (Van der Jeugd et al., 2009; Winkler et al., 2017). Recently, however, an extremely fast range expansion, outside annual range, occurred in Pink‐footed geese (*Anser brachyrhynchus*): these expanded from Svalbard eastwards across the Barents sea and colonised Novaya Zemlya, where they rapidly increased (Madsen et al., 2023). Vagrancy (occurrence outside normal species range; Dufour et al., 2024), especially at sea (Geisler et al., 2022), may have initiated this colonisation. Complementarily, the new migration route may have established by social learning (Madsen et al., 2023), as goose species mix in flocks (Cohen & Satterfield, 2020) and new habits can spread quickly through a population (Oudman et al., 2020; Tombre et al., 2019). Specifically, Pink‐footed Geese have likely copied their new migration route from Taiga Bean Geese (*Anser fabalis fabalis*), which winter in Denmark within the range of Pink‐footed Geese (Piironen et al., 2022) and moult in Novaya Zemlya (Piironen et al., 2021).

This rapid expansion may be related to climate change, of which the role can be twofold: as facilitator (making new areas available) and as driver (making traditional areas less optimal). Novaya Zemlya is colder than Svalbard, and warming has recently facilitated nesting there (Madsen et al., 2023). On the other hand, Svalbard has also warmed up and geese may now lag behind spring onset there. To understand population dynamics, it is crucial to know how spring onset affects fitness in both areas. Pink‐footed Geese are capital breeders (Klaassen et al., 2017) and in general, spring onset can have opposite effects on different stages of geese’ reproductive cycle (Nolet et al., 2020). In the first stages of reproduction (egg laying, incubation), early spring onset has positive effects, as geese start breeding sooner after arrival, keep more of their capital resources for breeding and have more foraging opportunities. This leads to a higher breeding propensity and nesting success (e.g. Boom et al., 2023; Madsen et al., 2007; Reed et al., 2004), partly through increased nest attendance which reduces nest predation (Samelius & Alisauskas, 2001; Spaans et al., 2007). In the later stage (chick rearing), early spring onset has negative effects on gosling growth and survival, by a trophic mismatch (e.g. Doiron et al., 2015; Lameris et al., 2018; Ross et al., 2018). Slower growth may in turn lengthen the exposure to predation (Dmitriew, 2011), although predation generally also depends on predator abundance (Layton‐Matthews et al., 2020). Thus, the new and traditional breeding areas may favour different stages of reproduction and whether an individual benefits from breeding in the new area may depend on its amount of capital.

Here, we examine potential costs and benefits associated with breeding in the new area. We combine tracking data of 83 individuals with long‐term population censuses and an extensive mark‐recapture programme. We evaluate potential constraints by the advancement and predictability of spring along the traditional flyway, how geese have tuned their timing to these changes, how spring onset affects reproduction in both flyways, and whether birds in both flyways are morphologically different. We expect that geese breeding in Novaya Zemlya are better matched with the food peak than in Svalbard, but that breeding there requires more body stores from parents, to overcome the costs of breeding in a later spring.

## 2 METHODS

### 2.1 Study area

Traditionally, the studied population of Pink‐footed geese winters in Flanders (Belgium), Friesland (Netherlands) and Jutland (west/north Denmark) and migrates in spring via Trøndelag and Vesterålen (Norway) to Svalbard (Madsen et al., 1999). On this route, the geese increasingly winter in Jutland (Clausen et al., 2018) and stop less at Vesterålen (Tombre et al., 2013). The new spring route runs from Lolland‐Falster (southeast‐Denmark) via Örebro (Sweden) and Oulu (Finland) to Novaya Zemlya (Madsen et al., 2023, Figure 1). Some geese also take the new route from Jutland onwards, or use one or two new stopovers (Örebro, Oulu) but still breed in Svalbard. We therefore define two breeding areas (Svalbard, Novaya Zemlya) and three spring routes (NOSB, Norway‐Svalbard; FISB, Finland‐Svalbard; FINZ, Finland‐Novaya Zemlya).

**FIGURE 1:**
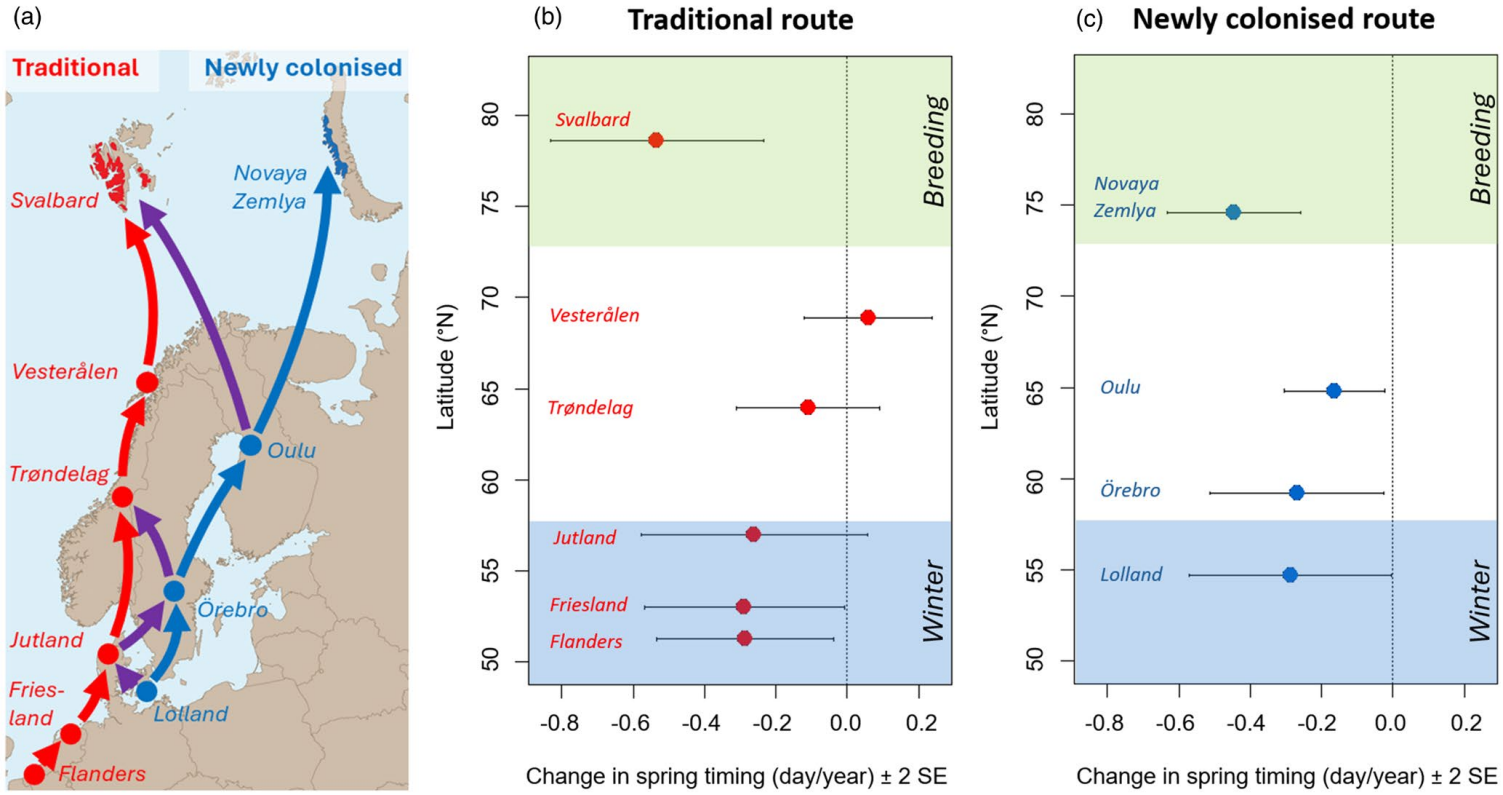
Contrasting rates of spring advance along the traditional and new migration route of Pink‐footed geese. (a) Geographic location of traditional (red) and new (blue) spring migration routes, including stopovers and breeding areas. Some birds use parts of both (purple). (b) The onset of spring was calculated based on growth degree days (GDD) as the moment at which the GDD accelerate fastest in spring. Trends in the onset of spring were tested for each location, over the period 1979–2022. The traditional route shows a steep advance in Svalbard but no change at northern stopovers, while on the new route, spring advanced less in Novaya Zemlya and did advance at the northern stopovers (c).

### 2.2 Catching, marking, tracking geese

We analysed two groups of geese: (1) individually marked geese (*n* = 5061 individuals) and (2) geese fitted with a tracker (*n* = 83). In all geese, we measured head length (total head + bill, with callipers, per 0.1 mm), wing length (with ruler, per mm) and body mass (with a spring scale, to nearest 50 g).

The first group was caught in 1990–2019 in winter, spring and summer, in the Netherlands (*n* = 21 individuals), Denmark (*n* = 3259), Norway (*n* = 1188), Finland (*n* = 14) and Svalbard (*n* = 558). The second group contains geese caught in Denmark in spring of 2003–2004 (*n* = 14 males, NOSB; Glahder et al., 2006) and 2011–2012 (*n* = 11 males, NOSB; Chudzińska et al., 2016) and newly tracked geese caught in Svalbard in summer 2018 (*n* = 33 females, 2 males, NOSB; Clausen et al., 2020), in Finland, spring 2018–2019 (*n* = 16 females, 5 males, FISB and FINZ; Schreven et al., 2021) and in Denmark, spring 2021 (*n* = 1 female, 1 male, NOSB; details in Supporting Information). In Svalbard, goslings and moulting adults were rounded up and caught in a netted corral, while at other locations the birds were caught by canon or clap net on agricultural fields. Capture and marking of geese in Svalbard was permitted by Mattilsynet (Norwegian animal research authority) to Aarhus University (reference no. 17/210528) and by the Governor of Svalbard (reference no. 17/01420–4). Capture on Isdammen was permitted by Longyearbyen Lokalstyre (reference no. 2018/347‐5‐X70). Capture and marking of geese in Finland was permitted by Etelä‐Suomen aluehallintovirasto to Aarhus University (ESAVI/1924/2018 and ESAVI/1880/2018) and by the Varsinais‐Suomen elinkeino‐, liikenne‐ ja ympäristökeskus (to Jorma Pessa, VARELY/551/2018).

### 2.3 Migration timing

Timing of migration and changes therein were studied with three datasets: resightings of individually neckbanded geese, counts of geese and tracking (details in Supporting Information).

Neckband resightings were analysed in Flanders (1992–2022, *n* = 22,682 observations), Friesland (1992–2022, *n* = 39,711), Jutland (1993–2004, *n* = 59,410), Trøndelag (1993–2022, *n* = 34,158), using the www.geese.org database (Ebbinge et al., 2020) and for Vesterålen (1991–2022, *n* = 18,937) the NINA database. We calculated several measures of migration timing for each location: for arrival, we calculated the first date by which over 5%, 50% or 95% of the seasonal total number of recorded neckbands had been observed. Similarly, for departure, we calculated the last date by which 95%, 50% or 5% of the seasonal total was still present based on the last observation of each goose. In Trøndelag, only the 5% arrival date was considered, since surveys allowed for assessing seasonal totals but were only frequent enough to study individual timing during the first part of the spring arrivals.

Field counts were analysed in Vesterålen and Oulu. In Vesterålen, geese were counted every 1–3 days from late April to late May, 1988–2022 (except 2013; Tombre et al., 2008). In Oulu, geese were counted during 1975–2022, first as national rarity, later with at least weekly counts. Sample size was sufficient only in 2000–2022. We calculated the same timing measures as with resightings.

Trackers registered ≥16 positions daily. For 2003–2004, migration timing was taken from Glahder et al. (2006). For 2011–2022, arrival and departure dates were calculated as the first and the last date on which a goose was on land at the stopover (within a radius of 50–100 km, Table S3). Stopovers were defined as the average GPS‐coordinates of tagged geese in the area (not flying, i.e. GPS‐speed <15 km/h; Geisler et al., 2022). Arrival date on the breeding grounds was defined as the first date on which a goose reached latitude >76.5640° (Svalbard) or longitude >51.4432° (Novaya Zemlya).

### 2.4 Timing and success of nesting

We identified nests with tracking data (2018–2022), following Schreven et al. (2021) using the ‘simultaneous GPS + ACC’ method, which combines data on geographic mobility and body motion. The median coordinates of locations where a goose was sitting still, on days when the goose was mostly sitting still, indicated a nest, when the goose had ≥3 days with >75% nest attendance. With this, we calculated per year the breeding propensity, that is proportion of geese attempting to nest. Egg‐laying date was defined as the first day with >75% nest attendance, as geese start egg laying immediately upon nest site selection (Inglis, 1977). Nests were classified as successful (≥1 egg hatched) when they had >75% daily attendance for 28–35 days (called ‘nesting success’; see also: Schreven et al., 2024). Hatching date (of successful nests) was taken as the last date with >75% attendance.

### 2.5 Brood survival and population reproductive output

GPS‐tracked geese from Svalbard and Finland were observed in the field in autumn and winter. We assessed whether a successfully nesting GPS‐tracked goose still had a brood (≥1 juveniles), called ‘brood survival’. Goose families stay together generally for half a year (see also: Gupte et al., 2019).

On population level, annual reproductive output was assessed by observers in late October. In Norway, Sweden, Denmark, Netherlands and Belgium, observers tallied numbers of juveniles and adults in flocks, which were averaged (weighted to number of geese present at stopovers) to estimate a population‐level proportion of juveniles. In late October, numbers in Sweden represented the Novaya Zemlya population, while other stopovers represented the Svalbard population (Madsen et al., 2023).

### 2.6 Spring onset

We used three measures of spring onset: based on temperature, snowmelt and vegetation green‐up.

These reflect different aspects and stages of spring conditions. The temperature‐based measure was defined as the date on which the growth degree days (GDD) accelerate fastest and is a good predictor of goose migration timing (Van Wijk et al., 2012). While GDD‐based spring may act as proximate cue for migration, snowmelt and green‐up may ultimately determine breeding performance, as these factors reflect the availability of nesting sites and food more directly.

GDD was calculated for the two breeding areas (each having six subareas) and eight stopovers (Tables S3–S5), for 1979–2022. We followed Boom et al. (2023), using R‐package ‘RNCEP’ (Kemp et al., 2012), taking linearly interpolated air temperature from the 2.5° × 2.5° gridded dataset (NCEP/DOE Reanalysis‐II; Kanamitsu et al., 2002). We did this for 1 Jan‐30 Sep, at 6 h temporal resolution. We averaged temperatures per day, to calculate GDD following Van Wijk et al. (2012). For breeding areas, subareas were averaged.

Snowmelt (available for 2000–2022) was calculated as the day on which half of the snow‐covered area had become snow‐free, derived from NDSI (normalised difference snow index). NDSI was obtained from MODIS satellite imagery (500 m resolution, c. 2 d temporal resolution; NASA, U.S.), with the Google Earth Engine (Gorelick et al., 2017) using R‐package ‘RGEE’ (Aybar et al., 2020; Versluijs, 2023), for the northern stopovers (Örebro, Oulu, Trøndelag, Vesterålen) and breeding areas. We followed Versluijs (2023) for each location and year (stopovers: 1 Jan‐30 Jun, breeding areas: 15 Mar‐15 Sep). For each spring, a generalised additive model (GAM) was fitted through NDSI data per pixel, using the R‐package ‘mgcv’ (Wood, 2015). Snowmelt was taken as the day on which GAM‐predicted NDSI‐values dropped below 0.42, indicating snow‐free conditions (following the Sentinel‐2 Level‐2A technical guide). Per spring and location (or breeding area), the median of pixels was taken as the date of snowmelt.

Vegetation green‐up (available for 2000–2022) was calculated as the day on which NDVI (normalised difference vegetation index) increased fastest (Wang et al., 2019). We again followed Versluijs (2023), based on MODIS data (500 m resolution, c. 2 d temporal resolution) for each location and year, as with snowmelt. For each spring, a GAM was fitted through the NDVI data per pixel. From GAM predictions, the day with largest NDVI increase was taken per pixel, which were then averaged per location and per breeding area.

### 2.7 Statistical analysis

Statistical tests were performed in R 4.2.1 (R Core Team, 2022). Temporal trends in spring onset were tested with linear models (LM) per location. We tested whether spring onset differed between routes (on stopovers and breeding areas), with LMs on within‐year differences. We also tested (LM) whether the interval in spring between last stopover and breeding area shortened over time.

The predictability of spring onset along migration routes was analysed by first detrending the spring onset data (including non‐significant trends). Residuals (data minus predicted values by temporal trends) were used for further analysis: we regressed (LM) spring at a location over spring at a previous location. We tested whether the predictability based on GDD had changed between 1979–1999 and 2000–2022, by including an interaction with period.

Migration timing was analysed with LM for resighting and count data, but for tracking data with linear mixed models (LMM, package ‘lme4’; Bates et al., 2015) including random intercepts of year and individual to account for non‐independence, using package ‘lmerTest’ to calculate p‐values (Kuznetsova et al., 2017). We tested temporal trends, differences between routes and relations with spring onset. With tracking data, we compared Svalbard and Novaya Zemlya geese on common stopovers.

For egg‐laying dates, we used LMM (random intercepts of year and individual). We compared Svalbard and Novaya Zemlya and tested whether egg‐laying date was related to spring onset. We also tested whether both breeding areas differed in relative laying date (laying date minus local spring onset) or in pre‐laying intervals (from arrival to egg laying). For the latter, we included only a random year intercept, as data were insufficient to allocate a random individual intercept.

Breeding propensity and nesting success (from tracks) were compared between Svalbard and Novaya Zemlya, using generalised linear mixed models (GLMM, binomial) with random intercepts of year and individual. We also tested how both variables related to spring onset, how breeding propensity related to relative arrival date (arrival date minus spring onset), and how nesting success related to relative laying date (laying date minus spring onset), while differentiating between Svalbard and Novaya Zemlya. Spring onset was taken at breeding area level (Svalbard, Novaya Zemlya), as pre‐breeding movements across both archipelagos may determine breeding performance.

Brood survival was compared between breeding areas (binomial GLMM, random intercepts of year and individual). We tested effects of spring onset, on the subarea level, as this is most relevant for chick growth and survival. We also tested effects of relative hatching date (hatching date minus spring onset). Total reproductive output (percentage juveniles in autumn) was compared (LM) between the two populations, and an effect of spring onset was tested.

Biometrics were analysed, of adults captured in Trøndelag (2015, 2016, 2017, 2022) and Oulu (2018, 2019). Per year, 1–5 captures were done between 24 April and 7 May. We compared wing length, head length and body mass between the traditional and new route, differentiating between the sexes, using LMMs with a random intercept of year. In case of body mass, we also corrected linearly for the capture date (day of year). Body condition (indicative of the amount of body stores) was defined as mass divided by expected mass (Green, 2001). Expected mass was defined as a model prediction given the goose's body size, based on regressions (LM) for sexes separately. Here, body size was taken as wing length, since mass‐wing regressions had higher R^2^ than mass‐head regressions (Table S6). We compared body condition between the routes (LMM, random intercept of year), and included an interaction with sex to test whether the difference varied between the sexes, while linearly correcting for the capture date.

## 3 RESULTS

### 3.1 Spring onset on stopovers and breeding areas

GDD‐based spring onset advanced significantly over 1979–2022 in Svalbard and Novaya Zemlya and on all stopovers except the traditional stopovers in Denmark and Norway. Spring advanced faster in the breeding areas than on the stopovers, and more uniformly on the new flyway (Figure 1, Table S7). Further, only in Svalbard snowmelt advanced significantly (period 2000–2022, *β* = −0.5675 ± 0.2123, *p* = 0.014).

GDD‐based spring onset was correlated between Svalbard and Novaya Zemlya over 1979–2022 (*r* = 0.44, *p* = 0.003), as were both snowmelt (*r* = 0.50, *p* = 0.015) and vegetation green‐up (*r* = 0.44, *p* = 0.034) during 2000–2022. Novaya Zemlya had later spring onset than Svalbard in 2000–2022 (GDD‐based within‐year difference: 15.97 days ± SE 1.98, *t* = 8.067, *p* < 0.001; snowmelt: 5.49 days ± SE 1.79, *t* = 3.074, *p* = 0.006; vegetation green‐up: 7.71 days ± SE 1.68, *t* = 4.592, *p* < 0.001).

The interval in GDD‐based spring between the last main stopover and the breeding grounds shortened significantly over 1979–2022 (Trøndelag‐Svalbard: −0.424 days/year ± SE 0.197, *t* = −2.149, *p* = 0.038; Oulu‐Novaya Zemlya: −0.282 days/year ± SE 0.104, *t* = −2.725, *p* = 0.009). Towards Svalbard, the interval was more variable (mean 10.9 ± SD 17.3 days) than towards Novaya Zemlya (mean 18.3 ± SD 9.3 days).

### 3.2 Predictability of spring along migration routes

**FIGURE 2:**
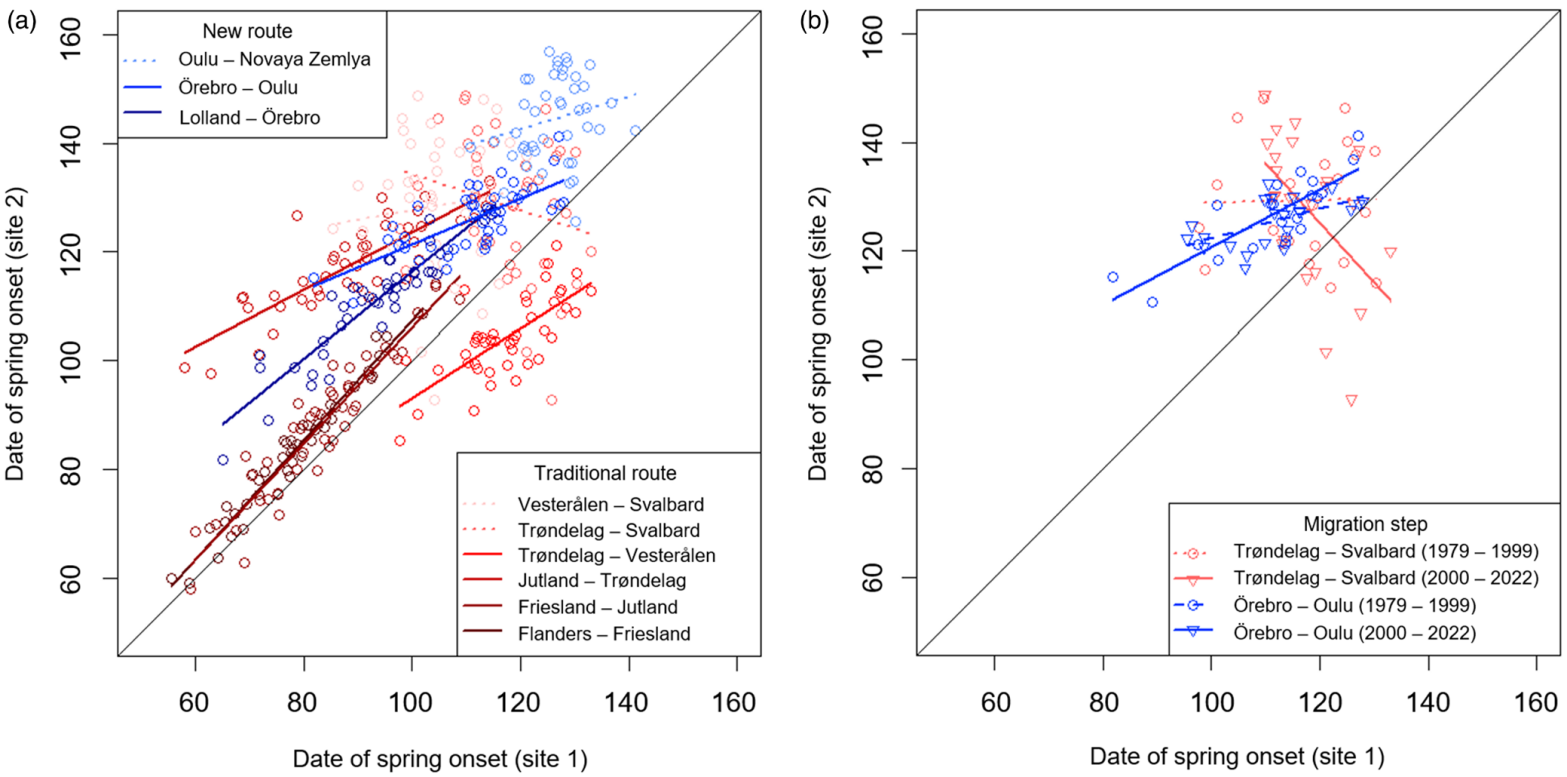
Predictability (spatial correlation) of spring onset for different migration steps and changes therein over time. (a) Spring onset was calculated based on GDD, and subsequently detrended for each site, over the entire period 1979–2022. Along both the traditional and new migration routes, spring onset in a certain site was regressed over the previous site on the route. Depicted are the detrended dates (residuals plus their mean spring onset over the whole period), as day of the year (120 = 30 April). Spring was correlated across mainland Europe, but not across the sea towards breeding areas. (b) When comparing two periods (1979–1999 versus 2000–2022), two migration steps showed a significant change in predictability of spring. Importantly, Trøndelag‐Svalbard changed from non‐correlated to negatively correlated. Both panels show non‐significant correlations as dotted lines, significant correlations solid or dashed. See also Table S8 for coefficients.

GDD‐based spring onset was spatially auto‐correlated over all migration steps during 1979–2022, except for over‐sea leaps from last stopovers to breeding areas (Figure 2, Table S8).

When comparing two periods (1979–1999 vs. 2000–2022), two changes were significant (Figure 2). Trøndelag‐Svalbard used to be uncorrelated (*p* = 0.92) before it became negatively predictable (*β* = −1.094 ± SE 0.394, *p* = 0.011) as the interaction year × period was significant (*p* = 0.019). Örebro‐Oulu used to be more predictable (interaction year × period: *p* = 0.037) but remained positively predictable in both periods (*p* < 0.001 and *p* = 0.004, Table S8).

### 3.3 Timing of migration

**FIGURE 3:**
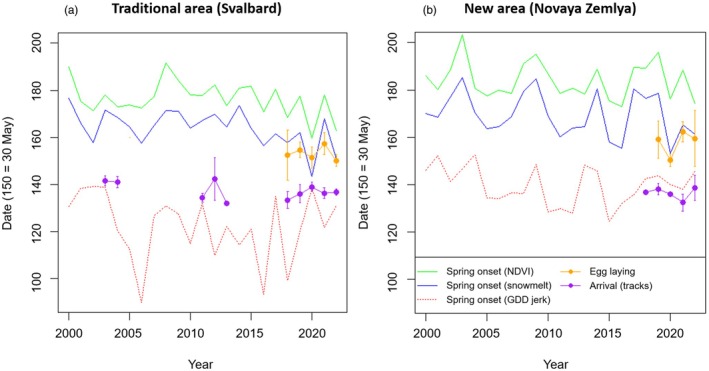
The timing of spring onset, arrival and egg laying in traditional and new breeding areas. For both the traditional (a) and newly colonised (b) breeding areas, the spring onset was defined with three measures: Based on GDD (moment of fastest temperature acceleration), snowmelt and NDVI (vegetation green‐up). The former measure was based on weather data reanalysis, the latter two on satellite imagery (analysing six subareas per area). The timing of arrival of and egg laying of Pink‐footed geese was studied with tracking devices (mean ± SD are given). Arrival date in Svalbard did not advance. In Svalbard, egg laying correlated with snowmelt and green‐up. While arrival did not differ between Svalbard and Novaya Zemlya, egg laying was later in Novaya Zemlya, but still longer before local green‐up. See also Table 1.

Goose migration timing did not change on wintering sites, but departure date in Jutland and arrival date in northern stopovers (Trøndelag, Vesterålen, Oulu) did (Figure 3, Table 1, Figure S3). In Vesterålen, also departure date advanced, while arrival in Svalbard showed a similar advance but not significantly so.

**TABLE 1:** Goose phenology on traditional and newly colonised stopovers and breeding areas.

Flyway	Area type	Location	Goose phenology temporal trend (day/year)	Goose phenology ~ spring onset (day/day)
Traditional	Stopover	Flanders	n.s. (Dep5_NB_, Dep50_NB_, Dep95_NB_)	Dep95_NB_ ~ GDD: 0.5839 ± 0.2609 (0.034)
		Friesland	n.s. (Dep5_NB_, Dep50_NB_, Dep95_NB_)	n.s.
		Jutland	Dep5_NB_: −2.976 ± 1.353 (0.053, n.s.) Dep50_NB_: −1.780 ± 0.218 (<0.001) Dep95_NB_: −0.822 ± 0.246 (0.007)	n.s.
		Trøndelag	Arr5_NB_: −0.9319 ± 0.0950 (<0.001) n.s. (Arr_tracks_, Dep_track_)	Arr5_NB_ ~ GDD: 0.4580 ± 0.2222 (0.049)
		Vesterålen	Arr50_count_: 0.1144 ± 0.0500 (0.029) Arr95_NB_: −0.1032 ± 0.0286 (0.001) Dep50_NB_: −0.0861 ± 0.0316 (0.011) Dep95_count_: −0.1314 ± 0.0232 (<0.001) Dep95_NB_: −0.1538 ± 0.0267 (<0.001) n.s. (Arr5_count_, Peak_count_, Dep50_count_, Arr5_NB_, Arr50_NB_, Dep5_NB_, Arr_track_, Dep_track_)	Arr5_NB_ ~ snow: 0.1205 ± 0.0405 (0.007) Dep5_NB_ ~ snow: 0.0494 ± 0.0222 (0.037)
	Breeding area	Svalbard	Arr_track_: −0.2457 ± 0.1257 (0.071, n.s.)	Lay_track_ ~ snow: 0.2609 ± 0.0819 (0.041) Lay_track_ ~ ndvi: 0.3160 ± 0.0774 (0.026)
Newly colonised	Stopover	Oulu	Arr5_count_: −0.7875 ± 0.1975 (<0.001) n.s. (Arr50_count_, Peak_count_, Dep50_count_, Dep95_count_)	Arr5_count_ ~ GDD: 0.8521 ± 0.3592 (0.027) Arr5_count_ ~ snow: 0.2182 ± 0.0859 (0.019) Arr50_count_ ~ GDD: 0.7905 ± 0.2476 (0.004) Arr50_count_ ~ snow: 0.1812 ± 0.0617 (0.008)
	Breeding area	Novaya Zemlya	‐‐ (Arr_track_, Lay_track_)	Arr_track_ ~ GDD: 0.7604 ± 0.2021 (<0.001)

*Note*: Per location, goose phenology was quantified with different variables (in column four, all are mentioned). These were determined based on neckband resightings (‘NB’), field counts (‘count’) or tracking data (‘track’). Migration was studied based on dates of arrival (‘Arr’) and departure (‘Dep’), with 5, 50 and 95 denoting the percentiles and ‘Peak’ the peak count date. Timing of breeding was defined by egg‐laying dates (‘Lay’). All these phenology variables were regressed over year (column four) and spring onset (last column). Spring onset was defined in three ways: Based on growth degree day acceleration (‘GDD’), snowmelt (‘snow’) or vegetation green‐up (‘ndvi’). Only (near‐)significant trends are given. Given are the slope estimate ± standard error and *p*‐value, ‘n.s.’ = not significant (*α* = 0.05), ‘‐‐’ = not tested, too few data. New stopovers Lolland and Örebro had too few data. For visualisation of data, see Figure 3 and Figure S3.

Migration timing was positively related to spring onset on several stopovers (Table 1). Important variables were GDD‐based spring (Flanders, Trøndelag, Oulu) and snowmelt (Oulu, Vesterålen). In breeding areas, arrival was related to spring onset (GDD‐based) in Novaya Zemlya only.

Departure date did not differ between Trøndelag and Oulu (GPS‐tracking, 2019–2022; *p* = 0.31, Figure 3, Table S3). In Oulu, geese departed earlier relative to snowmelt and GDD‐based spring than Trøndelag (on average 42 days vs. 79 days after snowmelt, *β* = 36.459 ± SE 1.009, df = 143.0, *t* = 36.139, *p* < 0.001; and 11 days vs. 22 days after GDD, *β* = 9.955 ± SE 0.635, df = 143.2, *t* = 15.673, *p* < 0.001) but later relative to green‐up (10 days vs. 4 days after NDVI, *β* = −8.272 ± SE 1.611, df = 143.3, *t* = −5.133, *p* < 0.001).

On some shared stopovers, timing differed between geese of Svalbard and Novaya Zemlya. In Jutland, geese from Novaya Zemlya stayed 3 weeks shorter than Svalbard geese did (LMM, *β* = 21.906 days ± SE 6.344, df = 40.0, *t* = 3.453, *p* < 0.001; Table S9) and departed c. 2.5 weeks earlier (LMM, *β* = 18.681 ± SE 2.872, df = 35.0, *t* = 6.504, *p* < 0.001). They also arrived and departed c. 2 weeks earlier in Örebro (LMM, arrival: *β* = 12.674 ± SE 4.782, df = 10.5, *t* = 2.650, *p* = 0.023; departure: *β* = 14.750 ± SE 6.157, df = 13.3, *t* = 2.396, *p* = 0.032) and departed c. 2 days later from Oulu than Svalbard geese (LMM, arrival: *p* = 0.513; departure: *β* = −2.223 ± SE 0.744, df = 18.2, *t* = −2.987, *p* = 0.008).

**FIGURE 4:**
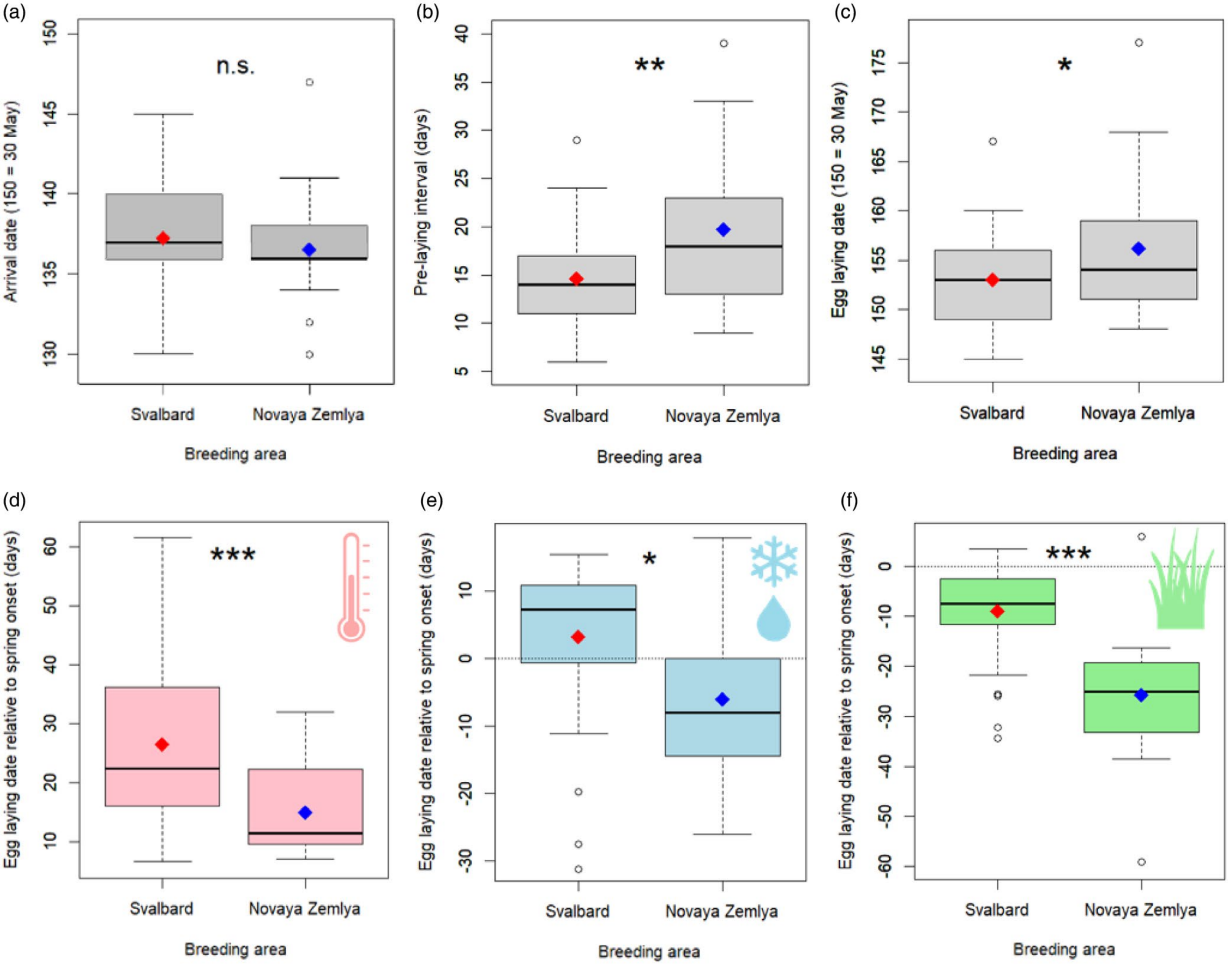
Location and timing of nesting in Svalbard and Novaya Zemlya. With GPS‐tracking (2018–2022), we determined arrival dates (a), the period between arrival and egg laying (b) and the egg‐laying dates (c), in traditional (Svalbard) and new breeding areas (Novaya Zemlya). The egg‐laying date relative to the local onset of spring was compared between both areas, with the onset of spring based on three measures: GDD acceleration (d), snowmelt (e) and vegetation green‐up (f). While arrival did not differ, pre‐laying intervals were longer in Novaya Zemlya and egg laying later. Still, relative to the onset of spring, egg laying in Novaya Zemlya was earlier. Significance was calculated in LMMs with random intercepts of year and individual, while overall boxplots and means (diamonds) are shown for visual clarity. Significance levels: n.s. = not significant, * = *p* < 0.05, ** = *p* < 0.01, *** = *p* < 0.001.

In Svalbard, arrival date did not advance significantly over 2003–2022 (LMM, *β* = −0.246 ± SE 0.126, df = 13.7, *t* = −1.955, *p* = 0.071; no long‐term data available for Novaya Zemlya). Arrival date did not differ between Svalbard (both routes combined) and Novaya Zemlya (LMM, Svalbard 0.5 days later, *p* = 0.59, Figures 3 and 4). Given the later spring onset in Novaya Zemlya compared to Svalbard, arrival in Novaya Zemlya was earlier relative to spring (on average 6 days before GDD, 28 days before snowmelt, 47 days before NDVI) than in Svalbard (10 days after GDD, 19 days before snowmelt, 32 days before NDVI; LMMs, GDD: *β* = 15.28 ± SE 0.83, df = 140.2, *t* = 18.499, *p* < 0.001; snowmelt: *β* = 10.96 ± SE 0.76, df = 41.1, *t* = 14.390, *p* < 0.001; NDVI: *β* = 15.88 ± SE 0.73, df = 44.2, *t* = 21.786, *p* < 0.001).

### 3.4 Timing of breeding

In Svalbard, egg laying occurred earlier than in Novaya Zemlya (average ± SD, Svalbard: 1.1 June ±5.8 days, Novaya Zemlya: 4.8 June ±6.6 days; LMM, *β*_SB‐NZ_ = ‐3.546 ± SE 1.629, df = 21.7, *t* = −2.177, *p* = 0.041, Figure 4), in line with later springs in Novaya Zemlya. When comparing different years in Svalbard, earlier laying date was explained by earlier snowmelt (LMMs, *β* = 0.261 ± SE 0.082, df = 3.48, *t* = 3.185, *p* = 0.041) and green‐up (*β* = 0.316 ± SE 0.077, df = 3.01, *t* = 4.085, *p* = 0.026), but not by GDD (*p* = 0.51), but such relations did not exist in Novaya Zemlya (*p* > 0.34). When comparing different subareas (within years), egg‐laying date was not related to spring onset measures, neither in Svalbard nor in Novaya Zemlya (LMMs; *p* > 0.08, Figure S4).

Since arrival date was similar in Svalbard and Novaya Zemlya, the pre‐laying interval (from arrival to laying) was longer in Novaya Zemlya (19.7 days, range 9–39) than Svalbard (14.6 days, range 6–29; LMM, difference: 5.591 days ± SE 1.283, df = 69.0, *t* = 4.357, *p* < 0.001, Figure 4).

Since Svalbard and Novaya Zemlya differed more in spring onset (on average 5–16 days) than in laying date, laying in Novaya Zemlya occurred earlier relative to local spring (on average 15 days after GDD, 6 days before snowmelt, 26 days before NDVI) than in Svalbard (on average 27 days after GDD, 3 days after snowmelt, 9 days before NDVI; LMMs, GDD‐based: *β* = −8.858 ± SE 2.192, df = 18.0, *t* = −4.041, *p* < 0.001; snowmelt‐based: *β* = −10.411 ± SE 4.165, df = 21.9, *t* = 2.500, *p* = 0.020; NDVI‐based: *β* = −20.031 ± SE 3.857, df = 20.3, *t* = −5.194, *p* < 0.001). With these laying dates, hatching would occur longer after NDVI‐based spring in Svalbard (19–26 days) than in Novaya Zemlya (2–9 days; Figure 4). Of hatched nests, hatch dates were on average 29.1 June ± SD 3.0 (range 24 June‐8 July) in Svalbard and 3.8 July ± SD 9.6 (range 27 June–28 July) in Novaya Zemlya (LMM, *β* = 4.728 ± 2.332, df = 23.4, *t* = 2.027, *p* = 0.054).

### 3.5 Breeding performance

**FIGURE 5:**
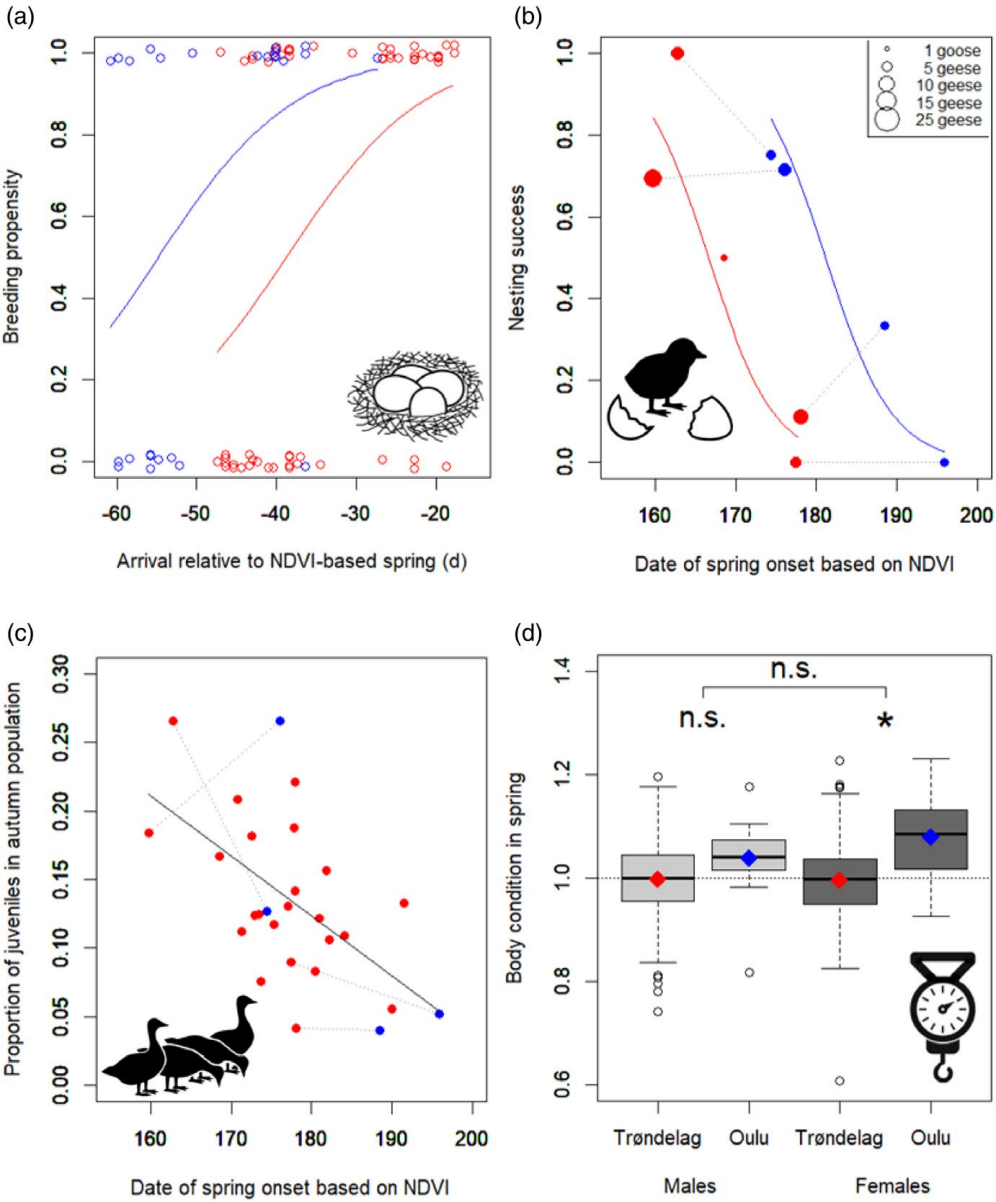
Breeding success and body condition in traditional and newly colonised flyways. In Svalbard (traditional breeding area) and Novaya Zemlya (new breeding area), we used GPS‐tracking to determine breeding propensity (chance to start breeding, a) and nesting success (chance of a nest to hatch at least one egg, b) of individual geese. In autumn, both subpopulations were surveyed to assess the proportion of juveniles (c). In spring, geese were captured in Trøndelag (traditional stopover) and Oulu (new stopover), to compare their body condition (i.e. mass divided by expected mass given wing length, d). The NDVI‐based spring indicates the moment of arctic vegetation green‐up. In panels (a, b), lines show model predictions of binomial GLMMs (on individual tracks, with random intercepts of year and individual), in panel (c) linear model predictions. For visual clarity, panel (b) shows annual means. In (b, c), data points of Svalbard and Novaya Zemlya in the same year are connected by grey dotted lines. Red = traditional areas, blue = newly colonised areas, n.s. = not significant, * = significant (*p* < 0.05).

Breeding propensity was similar between Svalbard (overall 59.7%, 37 out of 62 tracks) and Novaya Zemlya (overall 60.7%, 17 out of 28; GLMM: *p* = 0.97, Figure 5). Breeding propensity declined when spring started later (GLMMs; scaled NDVI: *β* = −1.364 ± SE 0.546, *z* = −2.495, *p* = 0.013; scaled snowmelt and GDD: *p* > 0.07; Figure S5). Corrected for spring onset, the breeding propensity still did not differ between breeding areas (based on NDVI: *β*_NZ‐SB_ = 1.925 ± SE 1.034, *z* = 1.862, *p* = 0.063; based on snowmelt and GDD: *p* > 0.28). The arrival date of geese relative to spring onset shows individual energy dynamics in a more direct and detailed way, even though arrival was relatively constant. Breeding propensity declined when geese arrived earlier before spring onset (NDVI‐based: *β* = 0.117 ± SE 0.042, *z* = 2.766, *p* = 0.006; snowmelt‐based: *β* = 0.096 ± SE 0.039, *z* = 2.431, *p* = 0.015; GDD‐based: n.s., Figure 5). Corrected for this effect, breeding propensity was higher in Novaya Zemlya than Svalbard (NDVI‐based: *β*_NZ‐SB_ = 1.801 ± SE 0.907, *z* = 1.986, *p* = 0.047; snowmelt and GDD: n.s.).

Nesting success did not differ between Svalbard (18 out of 36) and Novaya Zemlya (9 out of 17; GLMM, *p* = 0.99). Similar to breeding propensity, nesting success declined with later spring onset (GLMM: based on scaled NDVI: *β* = −2.649 ± SE 0.896, *z* = −2.955, *p* = 0.003; based on scaled snowmelt: *β* = −2.328 ± SE 1.039, *z* = −2.240, *p* = 0.025; based on scaled GDD: n.s.). Corrected for this effect, nesting success was higher in Novaya Zemlya than Svalbard (based on scaled NDVI: *β* = 3.495 ± SE 1.589, *z* = 2.199, *p* = 0.028; other measures n.s., Figure 5). Nesting success did not relate to relative laying date (laying date relative to local spring onset; all: *p* > 0.21), while correcting for breeding area.

Of successfully nesting geese, the proportion which still had their juvenile brood in autumn did not differ between Svalbard (8 out of 16, 2 unknown) and Novaya Zemlya (6 out of 8, 1 unknown; GLMM, *p* = 0.474). No significant effects of spring onset and relative timing of hatching on this proportion were found (all: *p* > 0.05).

The population juvenile percentage in autumn was higher when the spring had started earlier (LM, NDVI‐based: *β* = −0.0046 ± SE 0.0013, *t* = −3.491, *p* = 0.002; snowmelt‐based: *β* = −0.0049 ± SE 0.0012, *t* = −3.960, *p* = 0.001; GDD‐based: n.s.), while it did not differ between the Svalbard and Novaya Zemlya subpopulations in the same models (*p* > 0.50, Figure 5).

Overall, the proportion of successfully nesting geese (breeding propensity × nesting success) decreased from 0.74 in early springs, to 0.02 in late springs. This was a steeper decline than the proportion of juveniles in autumn, which decreased from 0.21 in early springs, to 0.05 in late springs.

### 3.6 Body size and condition

On the new route (Oulu), geese were generally larger and heavier than on the traditional route (Trøndelag; Table 2). The difference in head length (LMM; *β*_Oulu‐Trøndelag_ = 2.65 mm ± SE 1.04, df = 16.8, *t* = 2.542, *p* = 0.021) did not vary with sex (interaction route × sex: *β* = 0.006 mm ± SE 1.10, df = 1022.0, *t* = 0.005, *p* = 0.996) while males had longer heads than females (*β*_M‐F_ = 5.99 mm ± SE 1.08, df = 1022.0, *t* = 5.547, *p* < 0.001). The wing length difference between routes was only significant in males (LMM; *β*_Oulu‐Trøndelag_ = 8.44 mm ± SE 3.33, df = 301.7, *t* = 2.534, *p* = 0.012) but the interaction route × sex was not significant (*β* = −5.63 mm ± SE 4.56, df = 1022.4, *t* = −1.235, *p* = 0.217), while males had longer wings than females (*β*_M‐F_ = 20.13 mm ± SE 0.83, df = 1023.2, *t* = 24.233, *p* < 0.001). Contrarily, the body mass difference between routes was only significant in females (LMM; *β*_Oulu‐Trøndelag_ = 288.8 g ± SE 115.3, df = 6.6, *t* = 2.505, *p* = 0.043) and again the interaction route × sex was not significant (*β* = −50.76 g ± SE 90.45, df = 1022.3, *t* = −0.561, *p* = 0.575), while males were heavier than females (*β*_M‐F_ = 228.6 g ± SE 16.5, df = 1021.2, *t* = 13.891, *p* < 0.001) and correcting for day of year (*β* = 9.9 g/d ± SE 7.1, df = 54.1, *t* = 1.395, *p* = 0.169).

**TABLE 2:** Biometrics of adult geese on traditional and new migration routes.

		Traditional route (Trøndelag)	New route (Oulu)		
		Svalbard‐breeding (NOSB)	All geese	Svalbard‐breeding (FISB)	Novaya Zemlya‐breeding (FINZ)
Head length	Males	104.3 ± 3.4 (93–113) *n* = 591	107.1 ± 3.5 (98–111) *n* = 16	107.0 ± 3.4 (98–111) *n* = 2	107.4 ± 3.4 (98–111) *n* = 13
	Females	98.3 ± 2.9 (89–106) *n* = 405	101.1 ± 3.3 (95.5–108.9) *n* = 17	99.4 ± 2.9 (95.5–103.4) *n* = 6	100.8 ± 1.8 (97.5–103.3) *n* = 9
Wing length	Males	451.4 ± 13.3 (413–486) *n* = 591	459.9 ± 11.3 (437–480) *n* = 16	469.0 ± 5.7 (465–473) *n* = 2	460.3 ± 10.0 (445–480) *n* = 13
	Females	431.3 ± 12.2 (392–463) *n* = 405	434.2 ± 13.7 (398–455) *n* = 17	431.2 ± 20.4 (398–455) *n* = 6	436.1 ± 10.2 (423–454) *n* = 9
Body mass	Males	3195.7 ± 274.4 (2450–4100) *n* = 592	3428.1 ± 268.9 (2750–3900) *n* = 16	3175.0 ± 601.0 (2750–3600) *n* = 2	3492.3 ± 190.2 (3200–3900) *n* = 13
	Females	2965.1 ± 254.1 (1780–3750) *n* = 405	3247.0 ± 279.2 (2600–3650) *n* = 17	3058.3 ± 308.9 (2600–3550) *n* = 6	3400.0 ± 198.4 (3000–3650) *n* = 9
Body condition	Males	1.00 ± 0.069 (0.74–1.20) *n* = 591	1.04 ± 0.076 (0.82–1.18) *n* = 16	0.93 ± 0.16 (0.82–1.04) *n* = 2	1.06 ± 0.053 (0.983–1.176) *n* = 13
	Females	1.00 ± 0.069 (0.61–1.23) *n* = 405	1.08 ± 0.076 (0.927–1.232) *n* = 17	1.03 ± 0.063 (0.927–1.106) *n* = 6	1.12 ± 0.068 (1.013–1.232) *n* = 9

*Note*: During capture in spring (late April to early May, 2015–2022), head and wing length (mm) and body mass (g) were taken. Body condition was defined as mass divided by expected mass based on wing length (sex‐specific linear regressions). All geese captured in Trøndelag were assumed to breed in Svalbard (confirmed for a subset with GPS tracking), while the breeding area of geese captured in Oulu was known by GPS tracking or by resightings in autumn (Novaya Zemlya geese stop in Finland‐Sweden, not in Norway). The group ‘All geese’ in Oulu includes geese with unknown breeding area (1 male, 2 females); thus, it has a larger sample size than FISB + FINZ. Geese on the new route were generally larger and also relatively heavier than geese on the traditional route (Trøndelag). For significance of differences between routes and sexes, see text.

On the new route, body condition (mass divided by expected mass based on wing length) was significantly higher than on the old route, in females (LMM; *β*_Oulu‐Trøndelag_ = 0.0912 ± SE 0.0338, df = 5.8, *t* = 2.694, *p* = 0.037, Figure 5), while this difference was not significantly different in males versus females (interaction route × sex: *β* = 0.0441 ± SE 0.0233, df = 1021, *t* = 1.893, *p* = 0.059), and body condition increased with capture date (*β* = 0.0049 ± SE 0.0019, df = 100.0, *t* = 2.465, *p* = 0.015).

## 4 DISCUSSION

We studied Pink‐footed geese following their recent extreme range expansion in the Arctic, to examine potential costs and benefits associated with breeding in the newly colonised area, Novaya Zemlya, compared to the traditional area, Svalbard. Firstly, arrival dates in Svalbard had not advanced, even though spring onset in Svalbard had advanced rapidly, where consequently nests hatched a few weeks after the vegetation green‐up. Surprisingly, early spring in Svalbard was recently correlated with late spring on Norwegian stopovers. Contrarily, on the new flyway, geese were able to time their arrival in line with spring onset in Novaya Zemlya and spring started later in Novaya Zemlya than Svalbard. Consequently, nests hatched sooner after green‐up, suggesting better chick‐rearing conditions in Novaya Zemlya. On the other hand, in both areas, early springs had positive effects on breeding propensity and nesting success. Still, despite later spring onset in Novaya Zemlya, breeding propensity and nesting success were similar between Svalbard and Novaya Zemlya. This may relate to the fact that geese on the new flyway were relatively heavier in spring, thus possibly carrying more body reserves to handle the longer pre‐laying intervals and harsher conditions in Novaya Zemlya.

### 4.1 Limited responses in migration timing to advancing spring onset

As shown in other studies, spring had advanced rapidly in the Arctic, and to larger extent in Svalbard than Novaya Zemlya, as Svalbard has one of highest rates of warming on Earth (Isaksen et al., 2022). Spring advanced less in southern sites, in line with arctic amplification (Cohen et al., 2014). In this case, the discrepancy on the traditional route was aggravated as spring did not advance in Norway at all, possibly due to influence from the nearby Gulf Stream (Seager et al., 2002). Thus, spring advancing more in northern than southern sites reduces the opportunity for migrants to follow a ‘green wave’, as the progression of spring is more and more synchronised across latitudes, rather resulting in a ‘green flash’ (e.g. Ma et al., 2018; Rozenfeld et al., 2021).

Another challenge to migratory birds stems from annual variation in spring conditions. Spatial correlations in spring onset allow birds to time migration in pace with the progression of spring. Spring was generally correlated across mainland Europe but not across the sea towards breeding areas (see also Kölzsch et al., 2015). Thus, not only does the sea act as a barrier for feeding opportunities, but also for the predictability of spring. As a result, a fixed migration schedule (e.g. a clock‐based mechanism as in passerines; Åkesson et al., 2017) could give the ‘best guess’ but would limit the ability of birds to respond to variation in environmental conditions (see also Duriez et al., 2009), especially the recent negative correlation in spring onset between traditional stopover and breeding area. Pink‐footed geese did time their migration in line with spring on several stopovers, resembling other geese following a green wave (Van der Graaf et al., 2006; Van Wijk et al., 2012). Important spring metrics were in some cases snowmelt but mainly GDD‐based spring, likely because this variable showed high spatial autocorrelation across Europe. Previously, Pink‐footed goose migration was also found to correlate with dates of temperature sums (Kuijken et al., 2005) and green‐up (Tombre et al., 2008) but defining the green‐up in agricultural areas alone rather than the whole landscape gives different results, as agricultural activities also affect greenness. On traditional spring stopovers (Jutland, Trøndelag), goose migration advanced even though spring did not (but see Tombre et al., 2008), suggesting other possible dynamics, such as competition in Jutland, where increasingly more geese winter (Clausen et al., 2018) in line with diet shifts (Kuijken & Verscheure, 2023). In Vesterålen, migration advanced while spring did not, possibly a result of fewer geese using this stopover due to human scaring activities and competition with Barnacle geese (*Branta leucopsis*, Tombre et al., 2013). Interestingly, arrival to Novaya Zemlya correlated with local GDD‐based spring. Apparently, geese on the new route do have information to adjust their timing to, possibly benefitting from spring being slightly correlated between mainland Europe and arctic Russia (Kölzsch et al., 2015). A better predictability of arctic spring may further help birds to cope with arctic warming. In general, arctic migratory birds are currently advancing their timing of arrival and egg laying (Lameris et al., 2021, 2025). In birds, an important mechanism for this was spending less time on stopovers, to speed up migration and catch up with the progression of spring (Lameris et al., 2025). However, this requires stopovers that allow for earlier fuelling and for accurately predicting the arctic spring onset.

As birds colonise different breeding areas, their annual cycle adaptations might also select for different reaction norms to environmental conditions en route. Tracking enabled us to show that Novaya Zemlya geese departed earlier from Jutland than Svalbard geese. This may relate to the earlier spring in Örebro than Trøndelag, to higher competition avoidance behaviour (Schreven, 2023) or to following Taiga Bean Geese. The latter depart early (average 28 Feb, range 29 Jan‐26 Mar, GPS‐tracking data A. Piironen and A.D. Fox; Piironen et al., 2022). Thus, temporal separation of migrant subpopulations on shared stopovers is possible even in social species.

### 4.2 Consequences of range expansion for breeding

Range expansion of Pink‐footed geese towards colder areas had already occurred within the Svalbard archipelago (Tombre et al., 2012), which was predicted under climate change (Jensen et al., 2008), but the colonisation of Novaya Zemlya represents an extreme case on an unforeseen scale. Generally, barriers limit bird community shifts (Marjakangas et al., 2023) but the (interspecific) social migratory behaviour of geese may increase the likelihood of such jumps (Cohen & Satterfield, 2020; Madsen et al., 2023).

The later spring in Novaya Zemlya enables geese to start breeding longer before spring than in Svalbard, as arrival dates were similar. This has the benefit that nests hatched sooner after green‐up in Novaya Zemlya (<1 week) than in Svalbard (2–3 weeks), even though egg laying was some days later in Novaya Zemlya than Svalbard. Thus, in Novaya Zemlya, goslings likely faced a reduced mismatch, as they require nutritious young vegetation (Doiron et al., 2015). Also in other taxa, dispersal into areas with later phenology was related to a smaller trophic mismatch for offspring (insectivorous passerines: Hušek et al., 2014). Still, we did not detect an effect of relative hatching date on brood survival, but this may be partly masked because brood survival combines brood size and chick survival. The comparison of early and late springs revealed a larger difference in the proportion of successfully nesting geese than in the proportion of juveniles in autumn. This indicates that, in early springs, there must be either a lower chick survival, smaller initial brood size or higher adult survival. The latter two are unlikely, as average brood sizes are quite constant over early and late springs (Madsen et al., 2007), and in other goose species, adult survival was insensitive to summer temperature (Van Oudenhove et al., 2014). Thus, chick survival was likely lowered when spring is early, like in other geese (Lameris et al., 2018; Ross et al., 2018). The cause could be, apart from a trophic mismatch, more intense competition. In earlier springs, brood‐rearing areas may get overcrowded, as more pairs breed successfully. Effects of mismatch and density‐dependent competition probably occur simultaneously, and are difficult to disentangle (Brook et al., 2015; Ross et al., 2018).

Breeding longer before spring onset also carries a cost, that is nesting in harsher conditions. Longer before spring onset, temperatures are lower and foraging opportunities more limited due to snow cover (Eischeid et al., 2023). The effect of this cost was apparent within both Svalbard and Novaya Zemlya: breeding propensity and nesting success declined with later spring onset, as in other geese (e.g. Boom et al., 2023; Reed et al., 2004; Spaans et al., 1998). In late springs, fewer geese have enough capital resources left to breed, as they lose more before nesting, and consequently make more recesses during nesting, which increases nest predation (Spaans et al., 2007). However, this cost was not apparent when comparing Svalbard with Novaya Zemlya, as breeding propensity and nesting success were similar despite later springs in Novaya Zemlya. This suggests that Novaya Zemlya geese are better able to bear the costs of breeding or that the costs are lowered. The latter is unlikely given the cold springs and later snowmelt in Novaya Zemlya, but the former is supported by our data. Female geese on the new route were relatively heavier than those on the traditional route. This mass difference between routes was substantial, as it equates to the amount required for egg laying or half of incubation (Drent et al., 2003; Madsen & Klaassen, 2006). Male body condition differed less between routes, suggesting that male body stores play a smaller role. Females lay eggs and incubate, thus may be more susceptible to processes favouring larger body stores, even though male nest defence also increases nesting success (Samelius & Alisauskas, 2001). In addition, geese in Novaya Zemlya had bridged longer pre‐laying periods than in Svalbard, and geese that bridge a longer pre‐laying interval are usually still more successful during nesting (Dalhaug et al., 1996).

At the population level, positive effects of early springs on reproduction outweighed potential negative effects, as the proportion of juveniles was higher in years with an early spring. This is a general pattern in geese (Morrissette et al., 2010; Nolet et al., 2013; Trinder et al., 2009; but see Clausen & Clausen, 2013) and is explained by the geese’ longevity which makes them prioritise future reproduction (own survival) over current reproduction when the current conditions are harsh.

Tagging itself may have influenced reproductive performance. However, accounting for this would only magnify the difference between Novaya Zemlya and Svalbard. Svalbard geese were tagged in summer and Novaya Zemlya geese in spring. Tagging in spring impacts breeding performance more, in the subsequent breeding season (Schreven et al., 2024).

### 4.3 Individual variation in the effect of climate change on reproduction

Individual differences in, for example quality, behaviour or morphology, may cause individuals to experience different costs and benefits of certain environmental conditions (e.g. Dingemanse et al., 2004). This will also determine whether a change in conditions (e.g. by climate change) has a positive or negative effect on reproduction, and whether dispersal to new areas will pay off, leading to the evolution of dispersal syndromes (Stevens et al., 2014).

Firstly, in Pink‐footed geese, individuals on the new route were larger than on the traditional route, possibly indicating that larger individuals better overcome costs of dispersal (e.g. Van der Jeugd, 2001). Wing length only differed significantly in males, suggesting that competitive ability is more important for males (Van der Jeugd, 2001). The small head length difference might also have resulted from birds in Novaya Zemlya growing up with a smaller trophic mismatch (waders: Van Gils et al., 2016) or less competition (Loonen et al., 1997).

**FIGURE 6:**
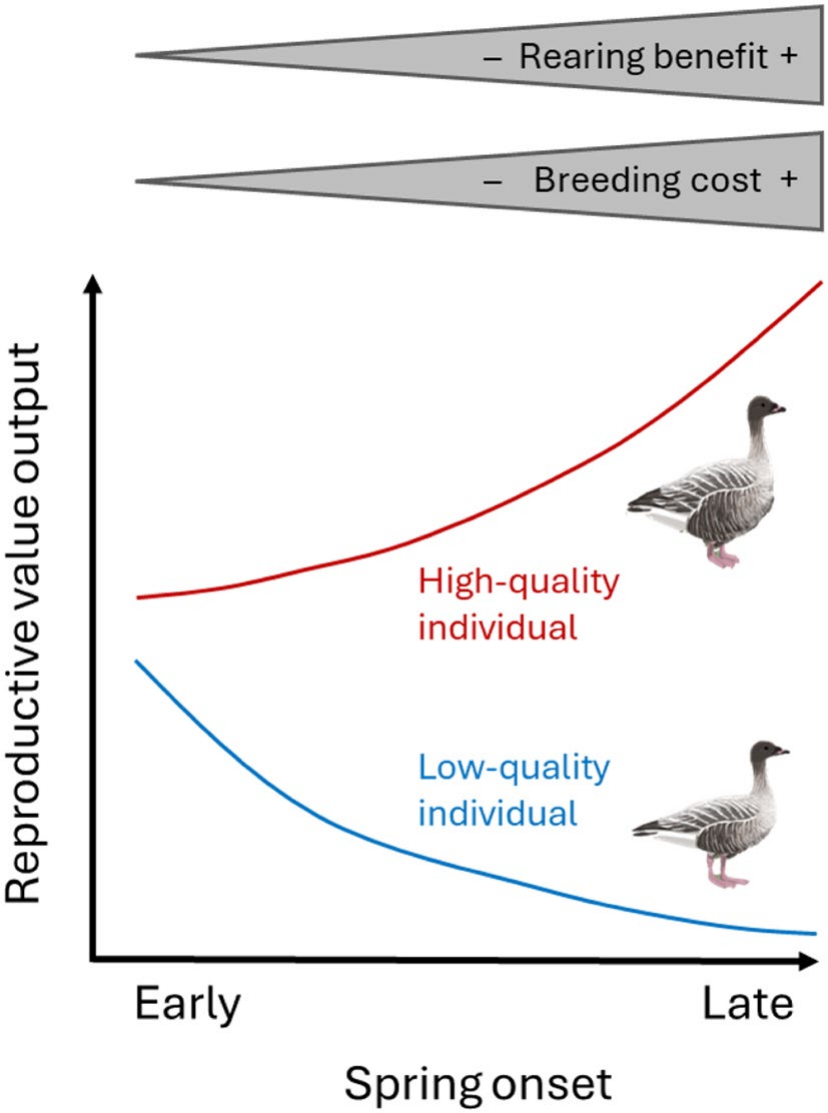
Hypothesised effect of individual quality on the relation between reproductive output and spring onset. The stage of breeding that limits an individual's reproductive output may differ with individual quality. Low‐quality individuals mainly manage to breed successfully in early springs (minimising breeding costs), while high‐quality individuals manage to breed in any spring but may benefit most from late springs (maximising rearing benefits, producing high‐quality offspring). This would drive mainly high‐quality individuals to colonise colder areas. Reproductive value output is the product of the reproductive output of the individuals and the reproductive value of their offspring. Breeding costs are the required capital resources (or, foraging abilities), rearing benefits are the reduced trophic mismatch and competition during growth.

Secondly, as outlined above, geese experience opposite effects of earlier springs during the early and late breeding stages: during early stages (laying, incubation) the effect is positive, but during late stages (chick rearing) negative (Nolet et al., 2020). This shows that a benefit of range expansion (i.e. a reduced mismatch) comes at a cost to parents (i.e. starting to breed in harsher conditions), which explains why Novaya Zemlya geese were relatively heavier. In such a system, we hypothesise that the driving and facilitating roles of climate change for range expansion are not homogenous in a population (Figure 6). Rather, individual quality would determine which stage is most limiting to an individual's reproduction. Low‐quality birds (i.e. with small body stores or foraging capacity) are expected to benefit most from earlier springs, as eased conditions enable them to breed at all, thus increasing offspring quantity. Contrarily, high‐quality birds (with large body stores or foraging capacity) can readily overcome costs of breeding and would benefit more from later springs, reducing the mismatch and competition for offspring, which increases offspring quality (a benefit expected to be less relevant to the output of lower‐quality birds). Growing up in good conditions has life‐long fitness benefits in birds, for example in fledging and adult size (Larsson & Forslund, 1991; Loonen et al., 1997), reproduction and survival (Monticelli & Ramos, 2012; Van De Pol et al., 2006). Given the highly skewed lifetime reproductive success in geese (e.g. Cooke et al., 1995), producing a few high‐quality offspring can pay off more than producing more lower‐quality offspring.

Therefore, we hypothesise that the role of climate change as driver and facilitator of range expansion acts on high‐quality individuals first, before it acts on lower‐quality individuals, as high‐quality individuals are expected to show more avoidance of areas where offspring is mismatched or faces large competition. Also in autumn, high‐quality pairs seek areas with less competition (Madsen, 2010). In capital breeders, individual quality and the amount of body stores are considered individual traits as they are repeatable over the years (waterfowl: Guillemain et al., 2013; Jaatinen et al., 2013). Still, Pink‐footed geese may partly also adjust fattening plastically, in anticipation of the later spring in Novaya Zemlya, and the lower competition on the new route could facilitate this (Schreven, 2023).

The generality of these dynamics can also be examined with species that have expanded their breeding range to warmer instead of colder areas. For example, Barnacle geese have started to breed in their wintering area, which eased nesting conditions (resulting in increased breeding propensity and nesting success compared to arctic breeding areas, Boom et al., 2023), while the mismatch of offspring increased (Van der Jeugd et al., 2009). Thus, breeding expansion towards warmer instead of colder areas may relate to benefits gained in the early stages rather than late stages of breeding (see also Nolet et al., 2020).

Apart from capital breeders, also income breeders may display such dynamics, as they face similar constraints acting on parents so that only high‐quality individuals (e.g. with better foraging abilities) are able to breed longer before spring onset (e.g. Drent & Daan, 1980). Also in short‐lived passerines, breeding early increases the costs of laying and incubation, with potential repercussions for adult survival (e.g. Visser et al., 2012). Acknowledging that climate change can affect the early and late breeding stages in opposite directions will help understand how variation in individual quality leads to heterogeneous effects of climate change within a population, which will in turn explain range expansion dynamics.

## AUTHOR CONTRIBUTIONS

Kees H.T. Schreven, Jesper Madsen and Bart A. Nolet conceptualised the study. Kees H.T. Schreven, Tom S.L. Versluijs, Michiel P. Boom, Jesper Madsen and Bart A. Nolet developed methodology. Kees H.T. Schreven, Fred Cottaar, Eckhart Kuijken, Jorma Pessa, Ingunn M. Tombre, Christine Verscheure, Jesper Madsen and Bart A. Nolet collected data. Kees H.T. Schreven analysed the data. Kees H.T. Schreven wrote the initial manuscript. All authors provided comments and contributed to the final manuscript. Kees H.T. Schreven, Jesper Madsen and Bart A. Nolet acquired funding. Jesper Madsen and Bart A. Nolet supervised.

## CONFLICT OF INTEREST STATEMENT

All authors declare that they have no competing interests.

## Supporting information


**Table S1.** Data sources of migration at stopovers.
**Table S2.** Sample sizes of independent tracks.
**Table S3.** Locations of stopover sites.
**Table S4.** Locations of subareas in Svalbard.
**Table S5.** Locations of subareas Novaya Zemlya.
**Table S6.** Allometry of body size measures in adult geese.
**Table S7.** Temporal trends of GDD‐based spring over time for different sites, 1979–2022.
**Table S8.** Predictability (spatial correlation) of spring onset for different migration steps, with spring based on GDD jerk, NDVI increase and snowmelt.
**Table S9.** Stopover duration of individually GPS‐tracked geese (2019–2022).
**Table S10.** Correlation between spring measures.
**Table S11.** Correlations of migration measures in Vesterålen.
**Figure S1.** Trends in spring onset in subareas in Svalbard and Novaya Zemlya.
**Figure S2.** Timing of snowmelt closely around nesting sites compared with the wider area.
**Figure S3.** The timing of spring onset and migration on different stopovers.
**Figure S4.** Egg‐laying date in relation to spatial variation in spring onset.
**Figure S5.** Breeding propensity, success and output in traditional and newly colonised areas.
**Figure S6.** Rates of climate change on the old and new migration routes, based on NDVI and snowmelt.
**Figure S7.** Difference in spring onset between the last stopover and the breeding area.

## Data Availability

Data are available from the DataverseNL Digital Repository, https://doi.org/10.34894/KLPQG9 (Schreven et al., 2025).
