# Supplementary material for: Arctic geese in newly colonised, colder breeding areas have higher spring body mass and breed earlier relative to the onset of spring
Source: J Anim Ecol. 2025 Nov 11;95(1):97–114. doi: 10.1111/1365-2656.70172 (PMC12775555; doi:10.1111/1365-2656.70172)

Supplementary materials

Arctic geese in newly colonised, colder breeding areas have higher spring body mass and breed earlier relative to the onset of spring.

Kees H.T. Schreven, Tom S.L. Versluijs, Michiel P. Boom, Fred Cottaar, Eckhart Kuijken, Jorma Pessa, Ingunn M. Tombre, Christine Verscheure, Jesper Madsen, Bart A. Nolet

**Supplementary Methods**

*Datasets on the timing of migration*

Sample sizes of different methods (neckband resightings, field counts, GPS/Argos tracking) are given per stopover site in Table S1.

The plastic neckbands were blue (1990-2005; n = 2493) or white (2007-2019; n = 2570) and both types were resighted frequently enough to derive migration routes and timing (Clausen et al. 2018). In these stopovers, geese were systematically and frequently observed, with a sufficient sample size. In Flanders, a marked goose was resighted on average 3.3 times per winter (range 1-23) and on average 235 geese were resighted per winter (range 69-497). Observations of GPS-tags were excluded in Flanders because observers targeted the GPS-tracks in the field there. In Friesland, a marked goose was resighted on average 4.5 times per winter (range 1-25) and on average 304 geese were resighted per winter (range 19-915). In Trøndelag, a marked goose was resighted on average 2.8 times per winter (range 1-23) and on average 400 geese were resighted per winter (range 44-895). In Vesterålen, a marked goose was resighted on average 2.4 times per spring (range 1-21) and on average 252 geese were resighted per spring (range 28-752). Örebro and Oulu were also monitored but numbers of neckbands were too low.

Our calculations of migration timing metrics slightly overestimate arrival date and underestimate departure date, but annual variation is assumed to reliably reflect temporal trends.

In Vesterålen, complete counts were conducted on average 12 times per spring (range 5-23) and the seasonal peak number was on average 9483 (range 2270-20,177). In whole Finland, Pink-footed geese were very rare migrants before 2000 (max. 24 geese per season), thus all observations have been stored in online databases (www.tiira.fi and www.birdlife.fi/havainnot/harvinaisuudet/rk/). Since 2000, targeted counts of geese were made in Oulu by a network of experienced observers at least once per week during April-May (peak migration time). Supplementary observations (e.g. first and last observation) were included based on the online databases and annual reports of the local bird watchers’ society (Pohjois-Pohjanmaan lintutieteellinen yhdistys/Aureola) for the whole period. Combined, the data mostly covered the whole counting area. In Oulu, during 2000-2022, on average 12 complete counts were made per spring (range 6-16) and the seasonal peak count was on average 1846 (range 78-5910).

The used transmitters (Table S2) were in 2003-2004 harness-attached backpack PTTs (Argos/GPS, Microwave Telemetry, Inc., USA), either 30 g battery-powered (n = 11) or 45 g solar-powered (n=3, 2004). These transmitters were programmed to operate in cycles (30 g: transmit 8 h, rest 15h; 45 g: transmit 10 h, rest 21 h). In 2011, 45 g solar-powered PTTs (Argos/GPS, Microwave Telemetry, Inc., USA) were used with the same attachment method (n=6), which were programmed to record one position every hour between 7:00–20:00 and one positions every 2 h at night, giving 18 positions per day. In 2012, 40 g PTTs (Argos/GPS, North Star Science and Technology, LLC, USA) were used with the same attachment method (n=5), programmed to record one position every hour between 6:00–19:00 and one position every four hours at night, giving 16 positions per day.

In 2018-2022, solar-powered GPS-GSM transmitter neckbands were used (type OrniTrack-N38, Ornitela UAB, Lithuania), weighing 38 g with inner diameter 38 mm. These were white with a black two-digit individual code, visible from a distance up to 900 m with a telescope in good conditions, and recorded a GPS-position and GPS-speed every 10-60 min depending on battery charge. The two geese caught on 6 March 2021 were not included in earlier studies, and were from catch site Ulvedybet, Gjølvej, Aabybro, Denmark (N 57°06', E 9°41'). Per year, only independent tracks were used (excluding partners and offspring when migrating together). Sample sizes lowered over the years due to mortality and tag failure. Per year, we tracked on average 11 NOSB-geese (range 1-26), 2 FISB-geese (range 1-4) and 6 FINZ-geese (range 2-10).

GPS-tracking allowed us to compare migration timing on both routes, and to compare migration timing of geese from different breeding areas on stopovers where they co-occur (sufficient data for Jutland, Örebro, Oulu). For other stopovers, we used trackers only when there were ≥ 6 years of tracking data (Trøndelag, Vesterålen).

*Sexing and measuring geese*

We sexed geese by cloacal examination in the field and validated this molecularly for the GPS-tagged geese in 2018-2022, following Fridolfsson and Ellegren (1999), using blood taken from a medial metatarsal vein, primer pair 2550F/2718R and the PCR-program of Griffiths et al. (1998), running results on a 2% agarose gel.

Head length was measured by KS and JM in Finland, and by JM in Norway. Wing length was measured by JM in both Finland and Norway. Although the small head length differences may be within the margin of measurement error, analysis on a different dataset (comparing Finland and Svalbard) measured by KS found similar results (Schreven 2023).

*Definition of subareas for analysis of spring onset*

The twelve breeding subareas were based on nesting sites of GPS-tracked birds. For temperature, each location was defined by midpoint coordinates. For vegetation greenness and snowmelt, each location (stopovers and breeding subareas) was defined by manually delineated polygons of the area that was visited most intensively by the GPS-tracked geese (excluding buildings, forests, water and permanent snow; Table S3-5). Örebro in spring 2020 had no snow and was omitted from snowmelt analysis. Polygons were created in Google Earth Pro (2021, version 7.3.4.8248) and imported in QGIS (version 3.16.6) to create shapefiles, which were then used for remote sensing the spring onset.

*Remote sensing the onset of spring*

Satellite data were obtained from the MODIS Terra Surface Reflectance Daily Global product (MOD09GA, v6.1, Vermote & Wolfe 2021). These data are atmospherically corrected and feature seven spectral bands, spanning from visible light to short-wave infrared, with a spatial resolution of 500 meters and near-daily global coverage (Vermote & Wolfe 2021). We used the MODIS quality band ‘state_1km’ to mask pixels that were classified as clouds according to the internal PGE11 cloud masking algorithm (Vermote et al. 2015).

The date of snowmelt was estimated per area of interest using a NDSI-based threshold approach (i.e. the Normalized Difference Snow Index, Dozier 1989, Dietz et al. 2012), where the NDSI was calculated as the normalized difference between the green and the short-wave infrared band:

$$NDSI= \frac{({Green}_{B04}-{SWIR}_{B06})}{({Green}_{B04}+{SWIR}_{B06})}$$

As turbid water surfaces such as rivers and lakes may also have high NDSI values (Hall et al. 1995), we excluded pixels corresponding to waterbodies (i.e. oceans, rivers, lakes and ponds) in the shapefiles for each area of interest (Table S3-5).

NDVI was calculated as the normalized difference between the near-infrared (NIR) and the red band:

$$NDVI= \frac{({NIR}_{B02}-{RED}_{B01})}{({NIR}_{B02}+{RED}_{B01})}$$

The GAMs were fitted using REML and the default thin plate regression splines (Wood 2023) without outlier filtering, as specifiable in the workflow of Versluijs (2023).

Snowmelt date was also assessed on a smaller scale (within 100 m) around each nest site, using Sentinel-2 satellite imagery (10 m resolution, on average 3 d temporal resolution; ESA). We used the same procedure as above, following Versluijs (2023).

**Supplementary Results**

*Correlations and trends in spring onset*

Per location, the different measures of spring onset were correlated in Örebro, Oulu and Novaya Zemlya (NDVI and snowmelt), and Oulu, Svalbard, Novaya Zemlya (GDD and snowmelt), but spring based on GDD and NDVI was uncorrelated (Table S10).

Within breeding areas Svalbard and Novaya Zemlya, spring was correlated between their six subareas (GDD-based 1979-2022: r>0.95, p<0.001; NDVI-based 2000-2022: r>0.54, p<0.05 for all pairwise correlations except two in Svalbard; snowmelt 2000-2022: r>0.48, p<0.05 for all pairwise correlations except one in Svalbard, see also Figure S1).

In 2000-2022, NDVI-based spring was only predictable from Flanders to Friesland (LM, β=0.5835 ± SE 0.2003, t=2.913, p=0.008), while snowmelt was only predictable from Trøndelag to Vesterålen (β=0.5728 ± SE 0.1596, t=3.588, p=0.002). Comparing GDD-based spring in 1979-1999 and 2000-2022, Trøndelag-Vesterålen remained positively predictable, while Vesterålen and Oulu turned into negative predictors of Svalbard (but non-significant, p>0.21, Table S8).

Snowmelt had advanced significantly in Svalbard only (-0.5675 d/y ± SE 0.2123, t=-2.674, p=0.014), while green-up had not changed significantly on stopovers nor breeding areas over 2000-2022 (Figure S4).

On the last main stopovers, spring started later on the new (Oulu) than traditional route (Trøndelag) in 2000-2022 (LM, within-year difference based on GDD: 7.228 d ± SE 1.207, t=5.99, p<0.001; based on snowmelt: 22.475 d ± SE 3.442, t=6.531, p<0.001; based on NDVI: n.s.).

During 2000-2022, on both routes, the interval in GDD-based spring between stopover and breeding area did not shorten significantly over time (all spring measures, Trøndelag-Svalbard: p>0.25, Oulu-Novaya Zemlya: p>0.41) and were longer when based on NDVI or snowmelt, c. 2-3 months (Figure S5).

*Correlation between different measures of migration timing*

In Trøndelag, the 5% arrival based on neckband resightings and tracking-based arrival were not significantly correlated (r=0.7295, df=4, t=2.133, p=0.10). However, in Vesterålen, measures based on field counts, neckbands, and tracking correlated significantly for arrival (4 out of 13 comparisons), and departure (5 out of 13 comparisons; Table S11).

In Flanders and Friesland, the departure of GPS-tags was rather late compared with the departure based on observations of marked birds. This is likely due to a decreasing observer effort towards the end of the winter (main text Figure 3).

The significant advance in both arrival and departure in Vesterålen (based on neckband resightings) is depicted in Figure S6.

*Breeding differences within Svalbard and Novaya Zemlya*

Arrival in Svalbard did not differ between geese from Norway or Finland (LMM, β_Norway-Finland_=1.52 ± SE 0.96 d, df=61.8, t=1.588, p=0.117). Within Svalbard, Finnish-ringed geese laid eggs earlier relative to the onset of spring (on average 30 d after GDD, 18 d before NDVI, 8 d before snowmelt) than Svalbard-ringed geese (on average 25 d after GDD, 6 d before NDVI, 7 d after snowmelt; LMMs, NDVI: β=10.735 ± SE 2.836, df=13.3, t=3.785, p=0.002; snowmelt: β=12.305 ± SE 4.074, df=14.1, t=3.021, p=0.009; GDD: n.s.), whereas their absolute laying date did not differ (LMM, β_FI-SB_=-0.3093 d ± SE 1.7826, df=16.0, t=0.173, p=0.864).

The areas in Svalbard where Finnish-ringed geese nested (i.e. north and central-east, Table S4) had later spring onset than areas of the Svalbard-ringed geese (i.e. central and west), based on GDD (LMM: 3.583 d ± SE 0.954, df=24, t=3.755, p=0.001), but not NDVI or snowmelt (p>0.06).

However, within Svalbard and Novaya Zemlya, laying date was not related to local spring onset (LMMs, GDD-based: β=0.1238 ± SE 0.1129, df=22.4, t=1.096, p=0.285; snowmelt-based: β=0.1242 ± SE 0.0767, df=29.2, t=1.619, p=0.116; NDVI-based: β=0.0996 ± SE 0.0866, df=27.3, t=1.150, p=0.260).

On a finer scale, within 100 m of nest sites, the spring onset did not differ between Svalbard and Novaya Zemlya (LMM; β_SB-NZ_: -6.62 ± SE 4.17 d, p=0.13). On Novaya Zemlya, spring around nests seemed to be a week earlier than in the wider landscape (LMM; β_Nest-Landscape_: -8.353 ± SE 4.557 d, df=20.4, t=-1.833, p=0.081), while there was no such difference in Svalbard, and this did not differ between breeding areas (LMM: β=-6.786 ± SE 4.706, df=23.9, t=-1.442, p=0.162, Figure S2).

*Biometrics*

Within geese on the new route, head and wing length did not differ between FISB and FINZ geese (LMMs, while correcting for sex; head: β_FINZ-FISB_=1.07 mm ± SE 1.29, df=27.0, t=0.829, p=0.42; wing: β_FINZ-FISB_=0.51 mm ± SE 5.50, df=27.0, t=0.093, p=0.93), but FINZ geese did have higher body mass (β_FINZ-FISB_=333.8 ± SE 105.0, df=27.0, t=3.178, p=0.004) and body condition (β_FINZ-FISB_=0.104 ± SE 0.0286, df=27.0, t=3.634, p=0.002) than FISB geese (Table 2).

**References**

Aybar, C., Wu, Q., Bautista, L., Yali, R., & Barja, A. (2020). rgee: An R package for interacting with Google Earth Engine. Journal of Open Source Software 5: 2272.

Chudzińska, M. E., Nabe-Nielsen, J., Nolet, B. A., & Madsen, J. (2016). Foraging behaviour and fuel accumulation of capital breeders during spring migration as derived from a combination of satellite-and ground-based observations. Journal of Avian Biology 47: 563-574.

Clausen, K. K., Madsen, J., Cottaar, F., Kuijken, E., & Verscheure, C. (2018). Highly dynamic wintering strategies in migratory geese: Coping with environmental change. Global Change Biology 24: 3214-3225.

Dietz, A. J., Kuenzer, C., Gessner, U., & Dech, S. (2012). Remote sensing of snow – a review of available methods. International Journal of Remote Sensing 33: 4094–4134.

Dozier, J. (1989). Spectral Signature of Alpine Snow Cover from the Landsat Thematic Mapper. Remote Sensing of Environment 28: 9–22.

Fridolfsson A.K., & Ellegren H. (1999). A simple and universal method for molecular sexing of non-ratite birds. Journal of Avian Biology 30: 116–121.

Glahder, C. M., Fox, A. D., Hubner, C. E., Madsen, J., & Tombre, I. M. (2006). Pre-nesting site use of satellite transmitter tagged Svalbard Pink-footed Geese Anser brachyrhynchus. Ardea 94: 679-690.

Gorelick, N., Hancher, M., Dixon, M., Ilyushchenko, S., Thau, D., & Moore, R. (2017). Google Earth Engine: Planetary-scale geospatial analysis for everyone. Remote Sensing of Environment 202: 18-27.

Griffiths, R., Double M.C., Orr K., Dawson R.J. (1998). A DNA test to sex most birds. Molecular Ecology 7: 1071–1075.

Hall, D. K., Riggs, G. A., & Salomonson, V. V. (1995). Development of Methods for Mapping Global Snow Cover Using Moderate Resolution Imaging Spectroradiometer Data. Remote Sensing of Environment 54: 127–140.

Schreven, K.H.T. (2023). Geese colonising New Land: causes and mechanisms of range expansion in an Arctic-breeding migrant. PhD thesis, University of Amsterdam, Amsterdam, The Netherlands.

Vermote, E. F., Roger, J. C., & Ray, J. P. (2015). MODIS Surface Reflectance User’s Guide (no. version 1.4) (pp. 1–35). MODIS Land Surface Reflectance Science Computing Facility. http://modis-sr.ltdri.org

Vermote, E., & Wolfe, R. (2021). *MODIS/Terra Surface Reflectance Daily L2G Global 1km and 500m SIN Grid V061*. NASA EOSDIS Land Processes Distributed Active Archive Center. https://doi.org/10.5067/MODIS/MOD09GA.061

Versluijs, T. S. L. (2023). RGEE_Snowmelt. https://doi.org/10.5281/zenodo.8229032

Wood, S. N. (2023). Package ‘ mgcv ’: Mixed GAM Computation Vehicle with Automatic Smoothness Estimation. R package version 1.9-1.

**Table S1. Data sources of migration at stopovers.** The sample size (number of years) for the assessment of migration timing of geese differs per method (neckband resightings, field counts, tracking) and stopover.

| **Stopover** | **Neckband resightings** | | | **Field counts** | | | **GPS/Argos tracking** | | |
| --- | --- | --- | --- | --- | --- | --- | --- | --- | --- |
|  | **Arrival** | **Departure** | **Arrival** | | **Departure** | **Arrival** | | **Departure** |  |
| Vesterålen | 32y | 32y | 34y | | 34y | 8y | | 8y |  |
| Trøndelag | 30y | - | - | | - | 6y | | 6y |  |
| Oulu | (too few) | (too few) | 48y | | 48y | 4y | | 5y |  |
| Örebro | (too few) | (too few) | - | | - | 4y | | 4y |  |
| Lolland | - | (too few) | - | | - | - | | (3y, too few) |  |
| Jutland | - | 12y | - | | - | - | | 4y |  |
| Friesland | - | 31y | - | | - | 4y | | 4y |  |
| Flanders | - | 31y | - | | - | - | | 4y |  |

**Table S2. Sample sizes of independent tracks.** We used tracking data of PTT-tags for 2003-2004 (Glahder et al. 2006) and 2011-2013 (Chudzińska et al. 2016) and GPS-tags that were newly deployed for this study (2018-2022). We report only independent tracks here; a tracked partner or offspring traveling along were excluded. Repeated: indicates the number of individuals that was tracked already for at least one previous year. For the Finland-Svalbard route, the number of geese is given that took this actual route (different from Fig. 1 where FI-SB was defined based solely on the ringing year). Notes *: 4 switched here from the FI-SB route, including 1 offspring now independent; **: 1 switched here from the FI-SB route.

| **Year** | **Norway-Svalbard route** | **Finland-Svalbard route** | **Finland-Novaya Zemlya route** | **Total** |
| --- | --- | --- | --- | --- |
| 2003 | 7 | - | - | 7 |
| 2004 | 6 | - | - | 6 |
| 2011 | 6 | - | - | 6 |
| 2012 | 6 (3 repeated) | - | - | 6 (3 repeated) |
| 2013 | 1 (1 repeated) | - | - | 1 (1 repeated) |
| 2018 | - | 4 | 2 | 6 |
| 2019 | 26 (4 repeated) * | 4 (1 repeated) | 10 (2 repeated) | 40 (3 repeated) |
| 2020 | 19 (all repeated) ** | 1 (all repeated) | 7 (all repeated) | 27 (27 repeated) |
| 2021 | 15 (13 repeated) | 1 (all repeated) | 7 (all repeated) | 23 (21 repeated) |
| 2022 | 10 (all repeated) | 1 (all repeated) | 5 (all repeated) | 16 (16 repeated) |

**Table S3: Locations of stopover sites.** The radius around the coordinates was used to determine arrival and departure date in the stopover areas. Surface area is that of the polygon used to determine spring based on NDVI and snowmelt with satellite images (indicated by orange polygons; not for Kristianstad). Arrival and departure are given as calendar dates (day number of year). Only arrival dates of birds coming from the south are included, i.e. *from Flanders, **from Flanders, Friesland, Lolland, ***from Lolland. Site 5 (Kristianstad) was not analysed further, as this is a minor stopover. The white scale is 20 km long.

| Nr | Stopover site | Country | Latitude (°N) | Longitude (°E) | Radius used for calculation | Surface area (km^2^) | Mean arrival date (range) | Mean departure date (range) |
| --- | --- | --- | --- | --- | --- | --- | --- | --- |
| 1 | Flanders | Belgium | 51.24788 | 3.146997 | 50 | 95.5 | - | 32 (11-53) |
| 2 | Friesland | Netherlands | 52.99464 | 5.545542 | 50 | 45.1 | * 28 (14-44) | 33 (14-49) |
| 3 | Jutland | Denmark | 56.97479 | 9.294092 | 100 | 236.1 | ** 37 (2-69) | 88 (47-107) |
| 4 | Lolland | Denmark | 54.68278 | 11.51577 | 50 | 42.1 | - | 30 (1-60) |
| 5 | Kristianstad | Sweden | 55.88397 | 14.21842 | 50 | - | *** 60 (59-60) | 54 (39-68) |
| 6 | Örebro | Sweden | 59.22110 | 15.25215 | 50 | 38.6 | 76 (40-101) | 88 (67-128) |
| 7 | Oulu | Finland | 64.82003 | 25.50833 | 50 | 63.5 | 99 (83-108) | 133 (129-138) |
| 8 | Trøndelag | Norway | 63.92808 | 11.43773 | 100 | 36.5 | 97 (83-129) | 134 (115-141) |
| 9 | Vesterålen | Norway | 68.87615 | 15.52473 | 100 | 32.6 | 134 (129-139) | 136 (130-141) |


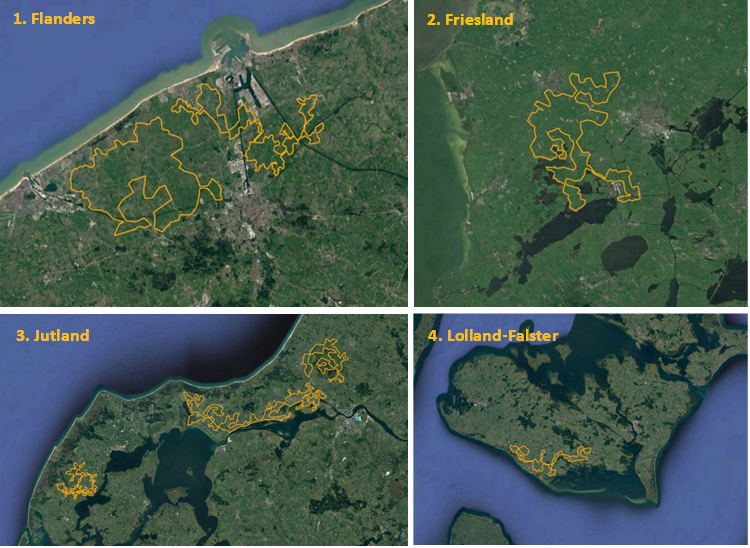

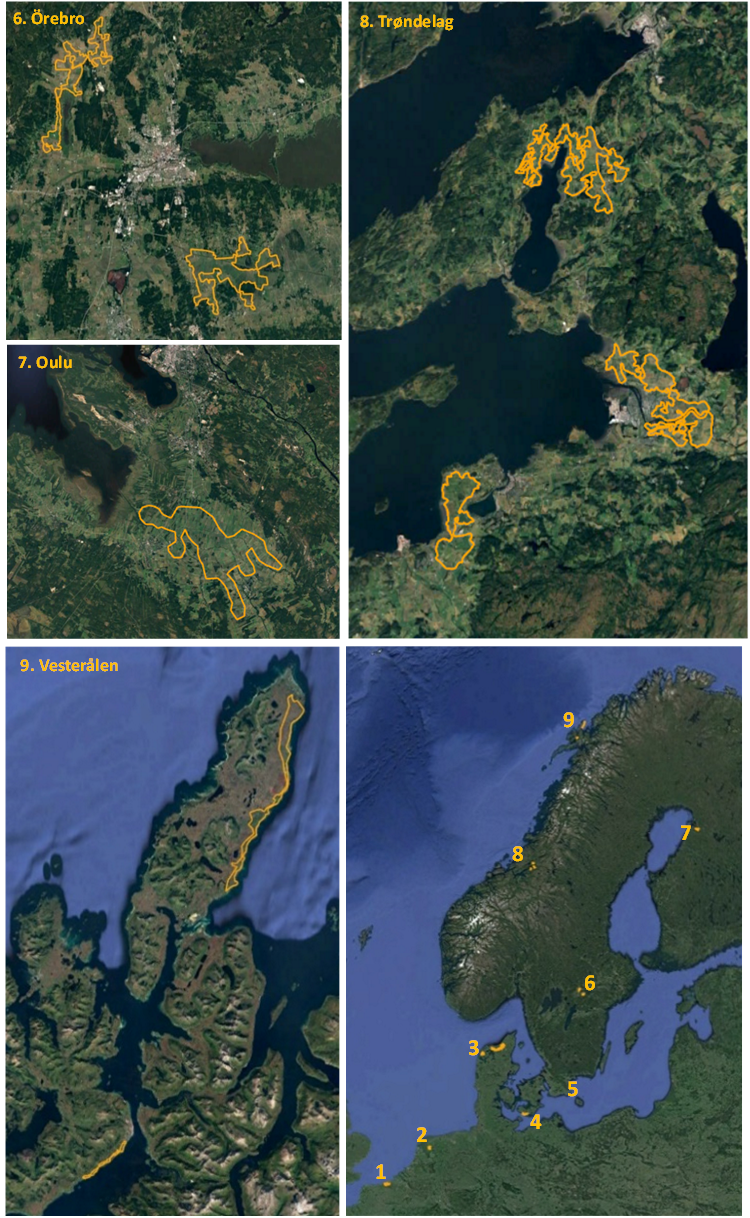


**Table S4. Locations of subareas in Svalbard.** Locations and area delineations were based on GPS-tracked geese. Placenames determined with <https://toposvalbard.npolar.no/>. Black scale is 20 km.

| **Nr** | **Name** | **Used by group** | **Latitude (°N)** | **Longitude (°E)** | **Surface area (km^2^)** |
| --- | --- | --- | --- | --- | --- |
| 1 | Reinsdyrflya | Finnish ringed | 79.79260 | 12.37008 | 71.6 |
| 2 | Daudmannsøyra | Svalbard ringed | 78.24444 | 13.07443 | 20.8 |
| 3 | Ekmanfjellet | Finnish ringed | 78.66999 | 14.32774 | 32.3 |
| 4 | Sauriedalen | Finnish ringed | 78.51817 | 15.40466 | 23.4 |
| 5 | Adventdalen | Svalbard ringed | 78.18354 | 15.77423 | 38.6 |
| 6 | Sassendalen | Finnish ringed | 78.31205 | 17.09271 | 32.0 |


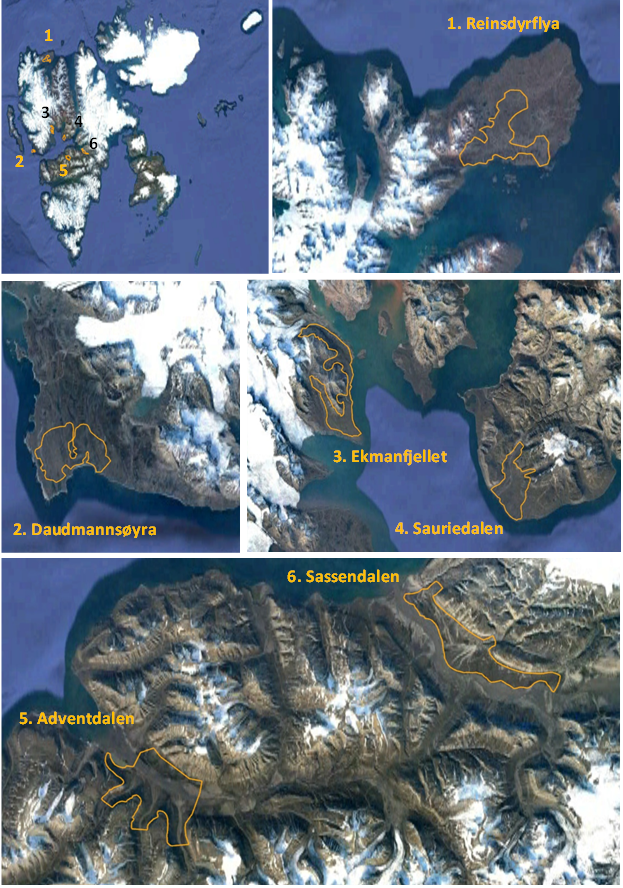


**Table S5. Locations of subareas Novaya Zemlya.** Locations and area delineations were based on GPS-tracked geese. Placenames based on <http://retromap.ru/1420012_73.390584,55.965728>. The black scale is 20 km.

| **Nr** | **Russian name** | **English name** | **Latitude (°N)** | **Longitude (°E)** | **Surface area (km^2^)** |
| --- | --- | --- | --- | --- | --- |
| 1 | п-ов крокодил | Krokodil | 75.74726 | 58.65703 | 11.6 |
| 2 | п-ов Адмиралтейства | Admiralteystva | 75.08020 | 55.94310 | 30.5 |
| 3 | тяжелый | Tyazhelyy | 74.90449 | 56.59742 | 34.2 |
| 4 | м. Макарова | Makarova | 74.65418 | 55.89477 | 30.6 |
| 5 | песцовая | Pestsovaya | 73.65184 | 55.42601 | 18.7 |
| 6 | залив мема | Zaliv Mema | 73.44747 | 55.93597 | 52.6 |


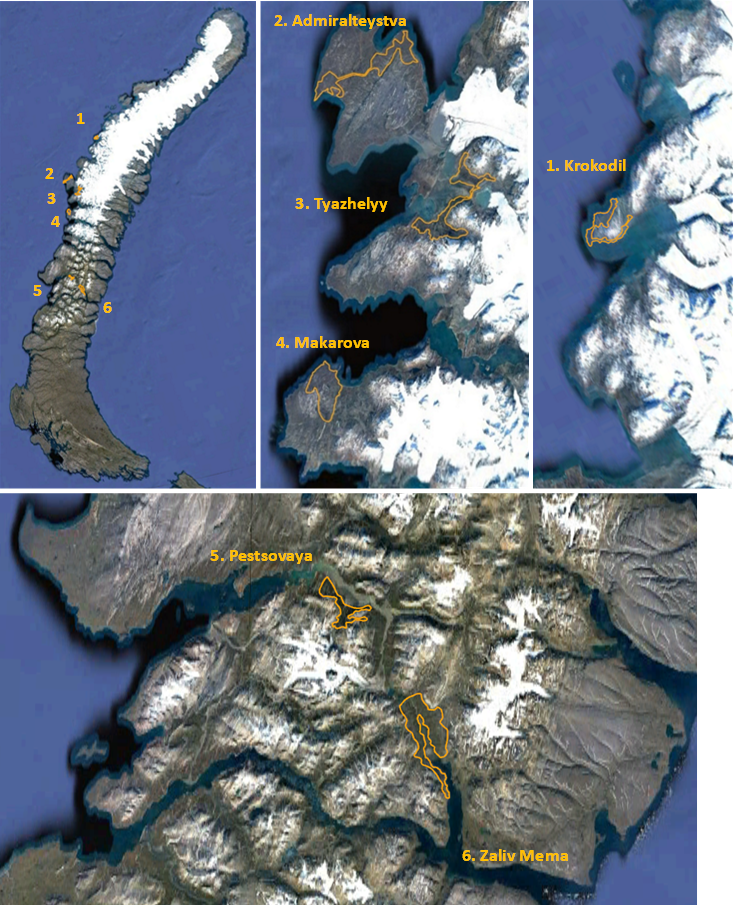


**Table S6. Allometry of body size measures in adult geese.** Geese were captured late April-early May in Norway and Finland. Per parameter (a or b), the estimate ± SE is given, with the p-value in brackets. These regression were used to calculate the expected weight given a body size, with which the body condition was calculated (weight / expected weight).

| **Sex** | **Formula** | **a** | **b** | **R-square** |
| --- | --- | --- | --- | --- |
| Males | Weight = a + b*wing | -2321.2 + 307.1 (<0.001) | 12.23 + 0.68 (<0.001) | 0.35 |
|  | Weight = a + b*head | -907.4 + 302.6 (0.003) | 39.37 + 2.90 (<0.001) | 0.23 |
| Females | Weight = a + b*wing | -2379.0 + 363.1 (<0.001) | 12.41 + 0.84 (<0.001) | 0.34 |
|  | Weight = a + b*head | -450.5 + 389.3 (0.248) | 34.82 + 3.95 (<0.001) | 0.15 |

**Table S7. Temporal trends of GDD-based spring over time for different sites, 1979-2022.** Results of linear regressions are given, with the intercept and effect size of year (and its standard error and p-value).

| **Area** | **Intercept** | **Slope estimate (d/y)** | **Slope SE** | **Slope p-value** |
| --- | --- | --- | --- | --- |
| Flanders | 652.875 | -0.2873 | 0.1241 | 0.026 |
| Friesland | 660.106 | -0.2883 | 0.1396 | 0.045 |
| Jutland | 609.852 | -0.2606 | 0.1590 | 0.109 |
| Lolland | 668.787 | -0.2875 | 0.1412 | 0.048 |
| Örebro | 650.020 | -0.2693 | 0.1222 | 0.033 |
| Oulu | 453.547 | -0.1637 | 0.0707 | 0.026 |
| Trøndelag | 335.849 | -0.1091 | 0.1003 | 0.282 |
| Vesterålen | -12.713 | 0.0585 | 0.0896 | 0.517 |
| Svalbard | 1194.524 | -0.5329 | 0.1488 | 0.001 |
| Novaya Zemlya | 1035.972 | -0.4457 | 0.0935 | < 0.001 |
| Adventdalen | 1165.191 | -0.5189 | 0.1458 | < 0.001 |
| Daudmannsoyra | 1177.260 | -0.5261 | 0.1519 | 0.001 |
| Ekmansfjellet | 1242.638 | -0.5569 | 0.1515 | < 0.001 |
| Reinsdyrflya | 1171.607 | -0.5197 | 0.1598 | 0.002 |
| Sassendalen | 1185.461 | -0.5280 | 0.1436 | < 0.001 |
| Sauriedalen | 1224.990 | -0.5480 | 0.1485 | < 0.001 |
| Admiralteytsva | 1150.878 | -0.5038 | 0.1016 | < 0.001 |
| Krokodil | 1085.461 | -0.4702 | 0.0936 | < 0.001 |
| Makarova | 1082.238 | -0.4691 | 0.0975 | < 0.001 |
| Pestjovaya | 918.167 | -0.3865 | 0.0903 | < 0.001 |
| Tyazhelyy | 1116.980 | -0.4865 | 0.0988 | < 0.001 |
| Zaliv mema | 862.108 | -0.3581 | 0.0879 | < 0.001 |

**Table S8. Predictability (spatial correlation) of spring onset for different migration steps, with spring based on GDD jerk, NDVI increase, and snowmelt.** Spring onset was first detrended, and then the second site was regressed over the previous site (linear model). The intercept, coefficient estimate, standard error and p-value are given. For GDD, two periods were analyzed (1979-1999 and 2000-2022) and the difference between periods (interaction year*period) is given as well.

1. For all three measures of spring onset over their entire time periods:

|  | Based on GDD jerk (1979-2022) | | | | Based on NDVI (2000-2022) | | | | Based on snowmelt (2000-2022) | | | |
| --- | --- | --- | --- | --- | --- | --- | --- | --- | --- | --- | --- | --- |
| **Migration step** | **Intercept** | **Slope** | **Slope SE** | **Slope**  **p-value** | **Intercept** | **Slope** | **Slope SE** | **Slope**  **p-value** | **Intercept** | **Slope** | **Slope SE** | **Slope**  **p-value** |
| Flanders – Friesland | -2.6948 | 1.1009 | 0.0356 | <0.001 *** | 17.1265 | 0.5835 | 0.2003 | 0.008 ** | - | - | - | - |
| Friesland – Jutland | -0.5564 | 1.0675 | 0.0613 | <0.001 *** | 90.4106 | 0.1916 | 0.1425 | 0.193 | - | - | - | - |
| Jutland – Örebro | 47.0358 | 0.7263 | 0.0389 | <0.001 *** | 51.1280 | 0.5191 | 0.3163 | 0.116 | - | - | - | - |
| Lolland – Örebro | 36.0174 | 0.8030 | 0.0499 | <0.001 *** | 59.6521 | 0.3877 | 0.4968 | 0.444 | - | - | - | - |
| Jutland – Trøndelag | 71.0342 | 0.5260 | 0.0537 | <0.001 *** | 111.8089 | 0.0698 | 0.2502 | 0.783 | - | - | - | - |
| Örebro – Trøndelag | 37.7551 | 0.7171 | 0.0614 | <0.001 *** | 109.4182 | 0.0915 | 0.1616 | 0.577 | 61.98393 | 0.1583 | 0.1901 | 0.415 |
| Örebro – Oulu | 79.2336 | 0.4211 | 0.0612 | <0.001 *** | 109.8802 | 0.1177 | 0.1457 | 0.428 | 79.74325 | 0.2314 | 0.1533 | 0.147 |
| Trøndelag – Vesterålen | 28.8976 | 0.6416 | 0.0960 | <0.001 *** | 98.2049 | 0.2024 | 0.1349 | 0.149 | 60.16715 | 0.5728 | 0.1596 | 0.002 ** |
| Trøndelag – Svalbard | 167.6701 | -0.3338 | 0.2231 | 0.142 | 184.5863 | -0.0684 | 0.0854 | 0.432 | 164.5213 | -0.0079 | 0.0628 | 0.902 |
| Vesterålen – Svalbard | 109.9944 | 0.1767 | 0.2548 | 0.492 | 176.7468 | -0.0025 | 0.1333 | 0.985 | 169.5956 | -0.0566 | 0.0665 | 0.404 |
| Oulu – Svalbard | 133.8158 | -0.0427 | 0.3246 | 0.896 | 175.6908 | 0.0062 | 0.0954 | 0.949 | 165.8674 | -0.0208 | 0.0797 | 0.797 |
| Oulu – Novaya Zemlya | 106.1960 | 0.3029 | 0.1986 | 0.135 | 187.2300 | -0.0251 | 0.1068 | 0.816 | 169.4798 | -0.0000 | 0.1077 | 1.000 |

1. Based on GDD jerk, testing the difference between two periods (1979-1999 and 2000-2022):

|  | Period 1979-1999 | | | | Period 2000-2022 | | | | Difference between periods | | |
| --- | --- | --- | --- | --- | --- | --- | --- | --- | --- | --- | --- |
| **Migration step** | **Intercept** | **Slope** | **Slope SE** | **Slope**  **p-value** | **Intercept** | **Slope** | **Slope SE** | **Slope**  **p-value** | **Difference in slopes** | **SE of difference** | **Difference p-value** |
| Flanders – Friesland | -6.3415 | 1.1455 | 0.0608 | <0.001 *** | 0.3243 | 1.0643 | 0.0414 | <0.001 *** | -0.0812 | 0.0720 | 0.267 |
| Friesland – Jutland | -6.2283 | 1.1311 | 0.0945 | <0.001 *** | 5.0574 | 1.0047 | 0.0808 | <0.001 *** | -0.1264 | 0.1237 | 0.313 |
| Jutland – Örebro | 45.2658 | 0.7499 | 0.0598 | <0.001 *** | 49.0844 | 0.7003 | 0.0516 | <0.001 *** | -0.0495 | 0.0795 | 0.537 |
| Lolland – Örebro | 33.1707 | 0.8384 | 0.0769 | <0.001 *** | 39.0016 | 0.7672 | 0.0658 | <0.001 *** | -0.0712 | 0.1015 | 0.487 |
| Jutland – Trøndelag | 65.0341 | 0.5960 | 0.0858 | <0.001 *** | 78.3722 | 0.4422 | 0.0637 | <0.001 *** | -0.1538 | 0.1079 | 0.162 |
| Örebro – Trøndelag | 26.6841 | 0.8162 | 0.0793 | <0.001 *** | 53.3340 | 0.5780 | 0.0925 | <0.001 *** | -0.2382 | 0.1220 | 0.058 |
| Örebro – Oulu | 67.8478 | 0.5295 | 0.0832 | <0.001 *** | 95.1885 | 0.2726 | 0.0832 | 0.0036 ** | -0.2569 | 0.1189 | 0.037 * |
| Trøndelag – Vesterålen | 21.1019 | 0.7086 | 0.1534 | <0.001 *** | 45.6577 | 0.4989 | 0.1045 | <0.001 *** | -0.2098 | 0.2086 | 0.321 |
| Trøndelag – Svalbard | 126.3444 | 0.0250 | 0.1020 | 0.920 | 256.5239 | -1.0942 | 0.3941 | 0.011 * | -1.1191 | 0.4570 | 0.019 * |
| Vesterålen – Svalbard | 94.1940 | 0.3366 | 0.2389 | 0.175 | 179.4413 | -0.4960 | 0.6576 | 0.459 | -0.8326 | 0.6502 | 0.208 |
| Oulu – Svalbard | 119.1889 | 0.0797 | 0.3373 | 0.816 | 179.4123 | -0.4122 | 0.6990 | 0.562 | -0.4918 | 0.7417 | 0.511 |
| Oulu – Novaya Zemlya | 107.7220 | 0.2906 | 0.2373 | 0.236 | 101.8786 | 0.3376 | 0.3933 | 0.400 | 0.0470 | 0.4573 | 0.919 |

**Table S9. Stopover duration of individually GPS-tracked geese (2019-2022).** The duration is given for two groups of geese (breeding in Svalbard versus Novaya Zemlya) as “mean ± SD (min-max) n=individuals/tracks” in days. The difference was tested per stopover in a LMM with random effects of year and individual, and is given as estimate ± SE in days, with the p-value in brackets.

| **Stopover** | **Svalbard-breeding geese** | **Novaya Zemlya-breeding geese** | **Difference** |
| --- | --- | --- | --- |
| Flanders | 30.83 ± 12.87 (10-52)  n=13/21 | 42.00 (-)  n=1/1 | -5.810 ± 10.317 (0.580) |
| Friesland | 12.00 ± 17.84 (0-48)  n=5/6 | 21.50 ± 30.41 (0-43)  n=2/2 | -9.500 ± 16.134 (0.574) |
| Jutland | 72.41 ± 21.48 (23-104)  n=28/68 | 51.88 ± 19.15 (13-80)  n=7/15 | 21.481 ± 5.679 (<0.001)*** |
| Örebro | 14.50 ± 13.72 (1-47)  n=5/10 | 11.52 ± 10.86 (0-33)  n=9/20 | 2.976 ± 4.543 (0.518) |
| Oulu | 34.00 ± 7.79 (25-41)  n=1/4 | 34.18 ± 6.22 (26-50)  n=9/21 | 0.017 ± 3.385 (0.996) |

**Table S10. Correlation between spring measures.** Correlation coefficients (r) and significance (p, in brackets) between different pairs of spring measures of the onset of spring, for each stopover and breeding area, 2000-2022.

| **Location** | **GDD and NDVI** | **GDD and snowmelt** | **NDVI and snowmelt** |
| --- | --- | --- | --- |
| Flanders | -0.1864 (0.395) | - | - |
| Friesland | -0.1568 (0.475) | - | - |
| Jutland | -0.1371 (0.533) | - | - |
| Lolland | -0.3474 (0.104) | - | - |
| Örebro | 0.1069 (0.627) | **0.6841 (<0.001)** | 0.0984 (0.663) |
| Oulu | -0.3797 (0.074) | **0.6807 (<0.001)** | **-0.4404 (0.035)** |
| Trøndelag | -0.1189 (0.589) | 0.3923 (0.064) | 0.0518 (0.814) |
| Vesterålen | -0.0537 (0.808) | 0.3049 (0.157) | 0.3665 (0.085) |
| Svalbard | 0.1135 (0.606) | 0.0993 (0.652) | **0.8640 (<0.001)** |
| Novaya Zemlya | 0.3208 (0.136) | **0.4391 (0.036)** | **0.8977 (<0.001)** |

**Table S11. Correlations of migration measures in Vesterålen.** For arrival and departure, the correlation coefficient (r) and the p-value (in brackets) are given, comparing migration timing as calculated from neckband resightings (1991-2022), field counts (1988-2012,2014-2022), and tracking data (2003-2004, 2011-2012, 2019-2022).

| **Arrival** | | Field counts | | | GPS-tracking |
| --- | --- | --- | --- | --- | --- |
|  |  | 5% arrival | 50% arrival | Peakdate | Arrival |
| Neckband resightings | 5% arrival | **0.718 (<0.001)** | **0.441 (0.021)** | 0.104 (0.577) | 0.355 (0.388) |
|  | 50% arrival | -0.113 (0.544) | **0.379 (0.036)** | **0.372 (0.040)** | 0.179 (0.672) |
|  | 95% arrival | -0.018 (0.924) | 0.025 (0.892) | 0.328 (0.072) | 0.107 (0.801) |
| GPS-tracking | Arrival | -0.143 (0.736) | 0.588 (0.125) | 0.123 (0.772) | - |

| **Departure** | | Field counts | | | GPS-tracking |
| --- | --- | --- | --- | --- | --- |
|  |  | 5% departure | 50% departure | Peakdate | Departure |
| Neckband resightings | 5% departure | **0.394 (0.028)** | 0.305 (0.096) | 0.217 (0.240) | -0.021 (0.961) |
|  | 50% departure | 0.424 (0.018) | **0.566 (<0.001)** | **0.443 (0.013)** | 0.212 (0.614) |
|  | 95% departure | 0.843 (<0.001) | **0.453 (0.010)** | 0.321 (0.079) | **0.708 (0.049)** |
| GPS-tracking | Departure | 0.627 (0.096) | 0.156 (0.713) | -0.339 (0.411) | - |

**Figure S1. Trends in spring onset on subareas on Svalbard and Novaya Zemlya.** Measures of spring onset (GDD jerk, NDVI increase, snowmelt) were generally correlated between subareas. Solid trendlines significant, dotted n.s.


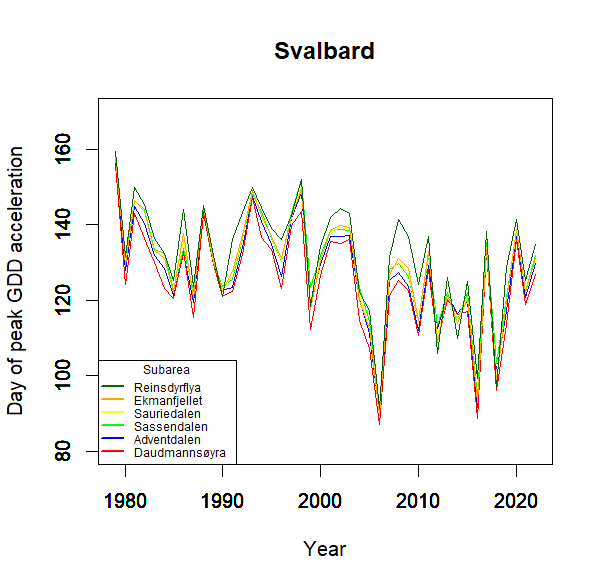

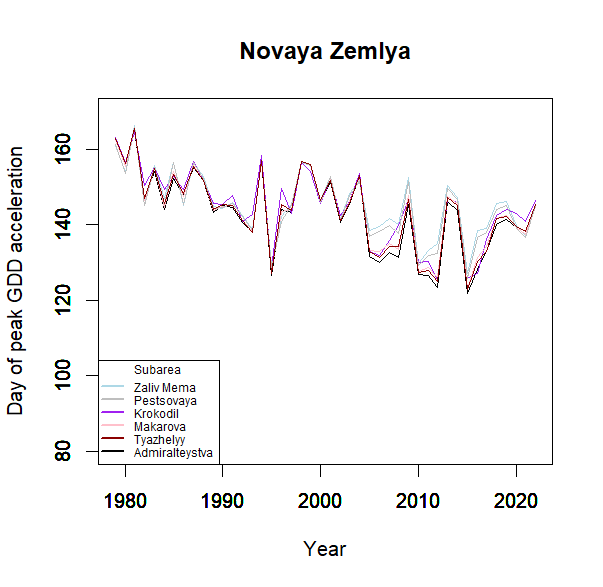

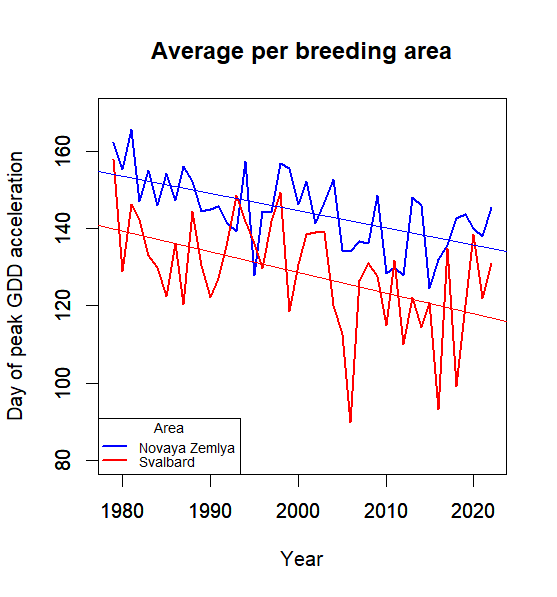


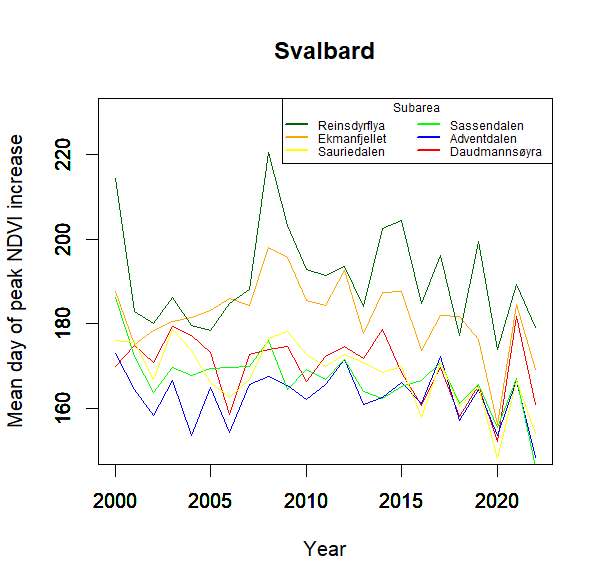

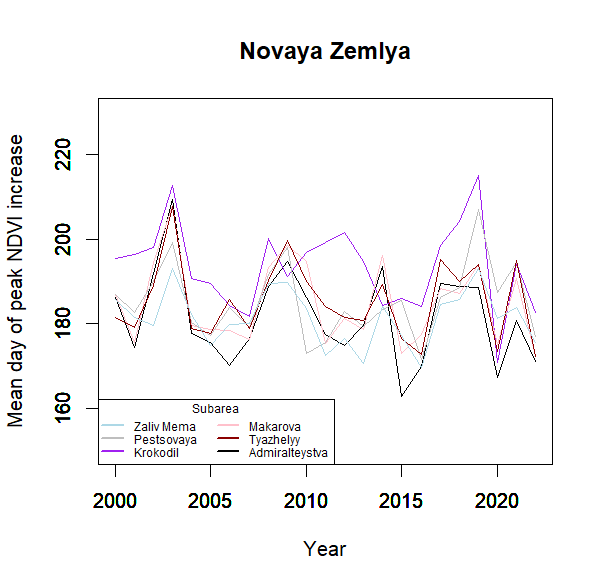

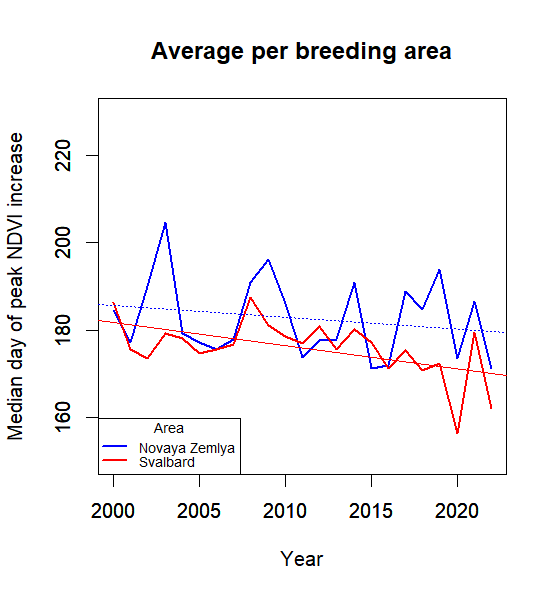


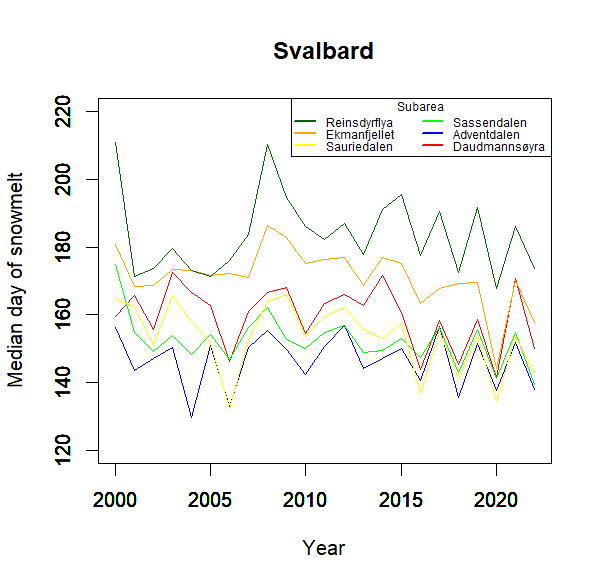

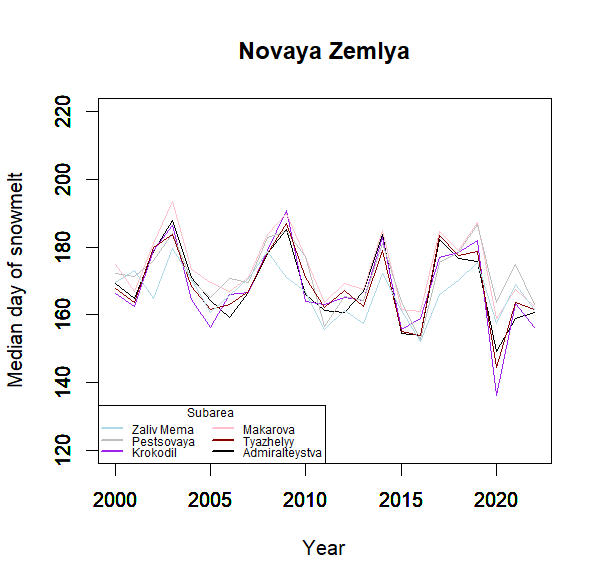

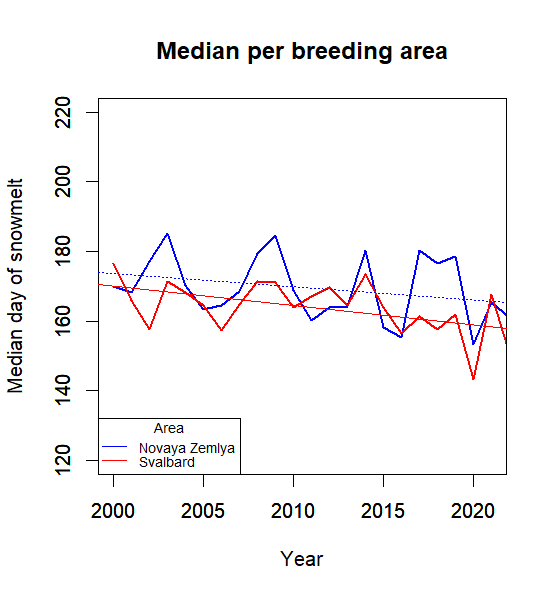


**Figure S2. Timing of snowmelt closely around nesting sites compared with the wider area.** During 2018-2022, the snowmelt date did not differ between Svalbard and Novaya Zemlya (A; whereas it did during 2000-2022). Closely (< 100 m) around nesting sites, the difference in snowmelt date was even smaller (B). The GPS-tracked geese (2018-2022) seemed to select nesting sites on Svalbard that were snow-free later than the wider area, whereas on Novaya Zemlya they seemed to select relatively early snow-free areas, but this difference was not significant (p=0.16; C).


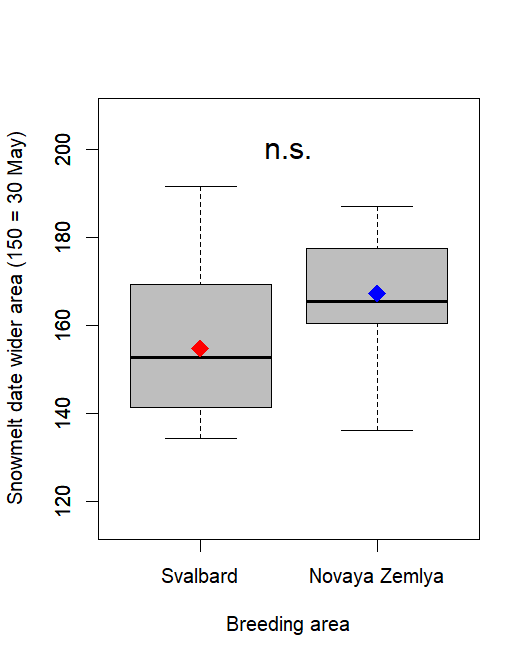

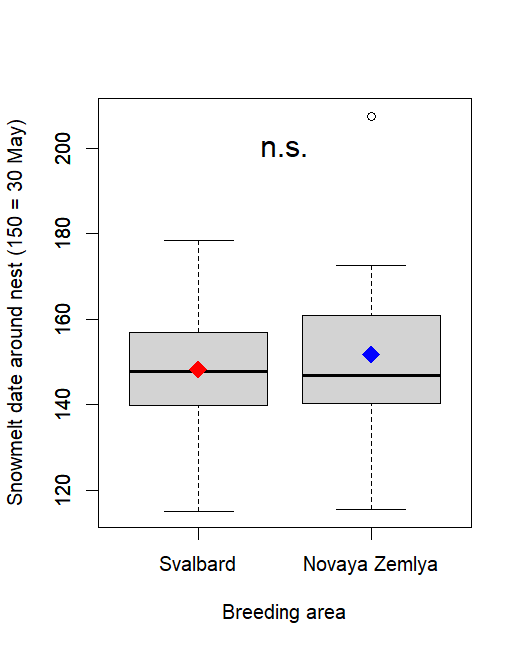

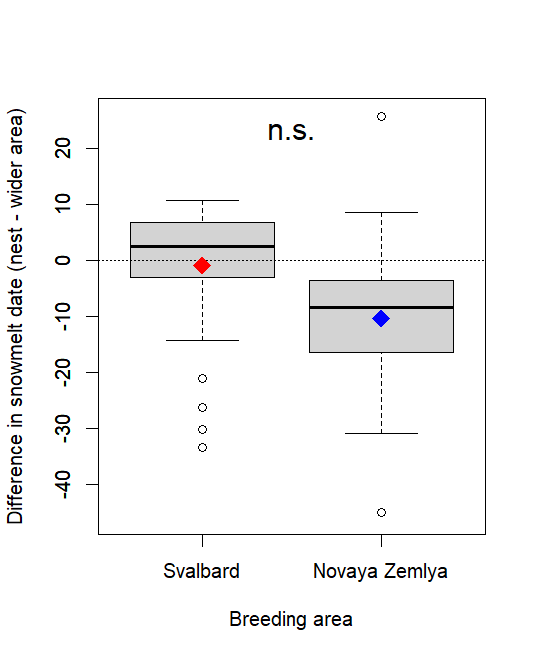


**Figure S3. The timing of spring onset and migration on different stopovers.** Per location, goose phenology was quantified based on neckband resightings (A, B, C, E; grey lines), field counts (F, H, grey shading) or tracking data (all panels A-H). Spring onset was defined in three ways: based on growth degree day acceleration (‘GDD’), snowmelt (‘snow’), or vegetation green-up (‘NDVI’). In panels C, D, H, timing is given for two groups of geese separately based on their breeding area (SB = Svalbard, NZ = Novaya Zemlya). In Oulu (G), only 2000-2022 was analysed (before 2000 were insufficient data). Vesterålen is depicted in panels F and G as it had too many data to display in one panel (data from GPS-tracking is overlaid in both, for comparison). For tracking data, mean ± SD is given. Legends are given per panel, but spring onset measures (green, blue, red lines) are given separately in the bottom right. See Table 1 for coefficients of trends.


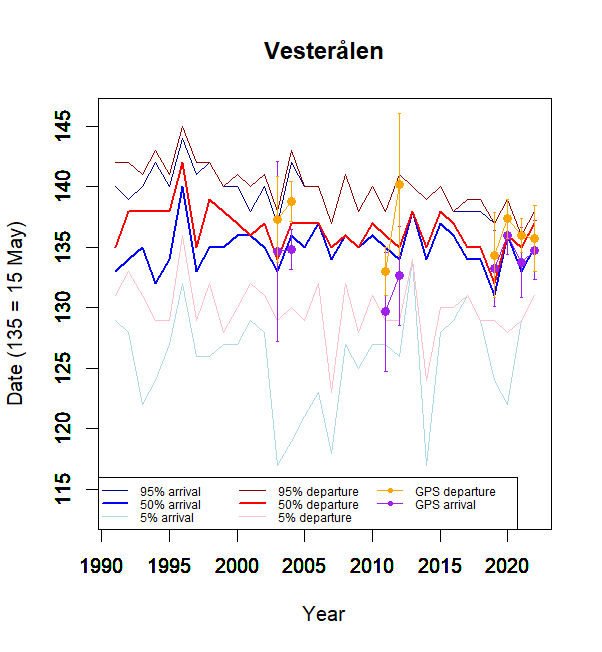

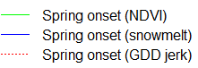

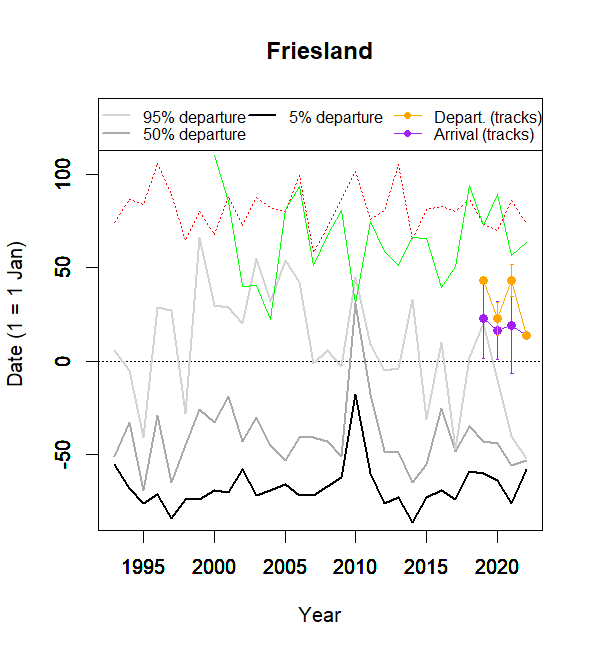

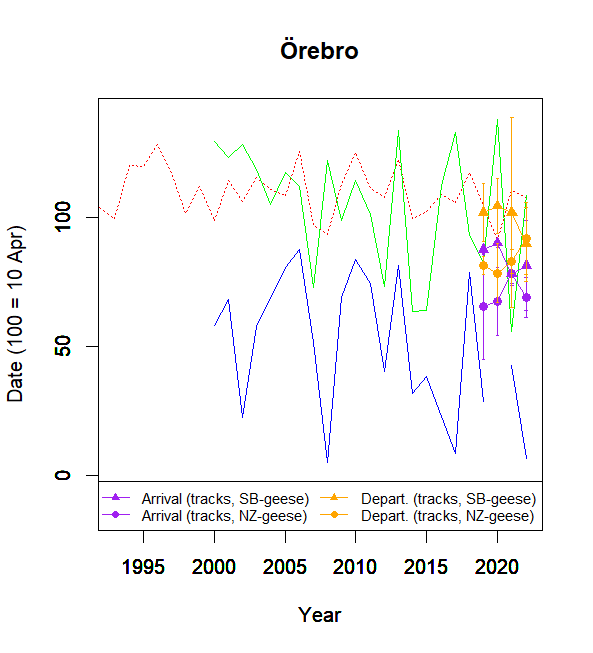

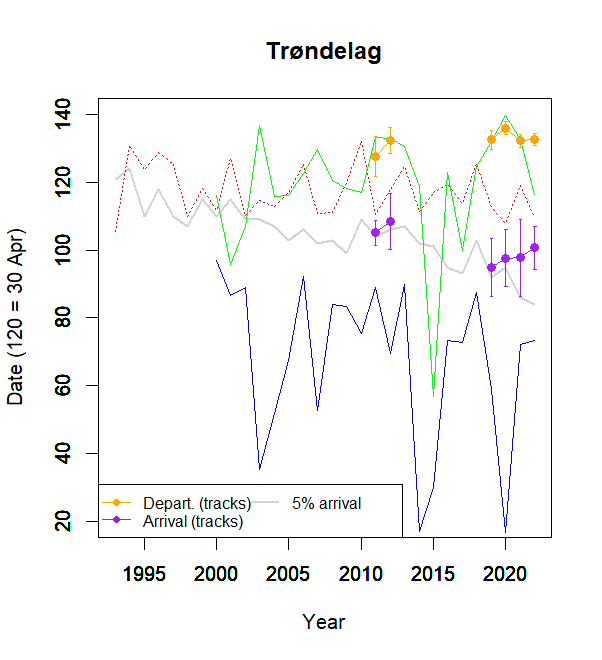

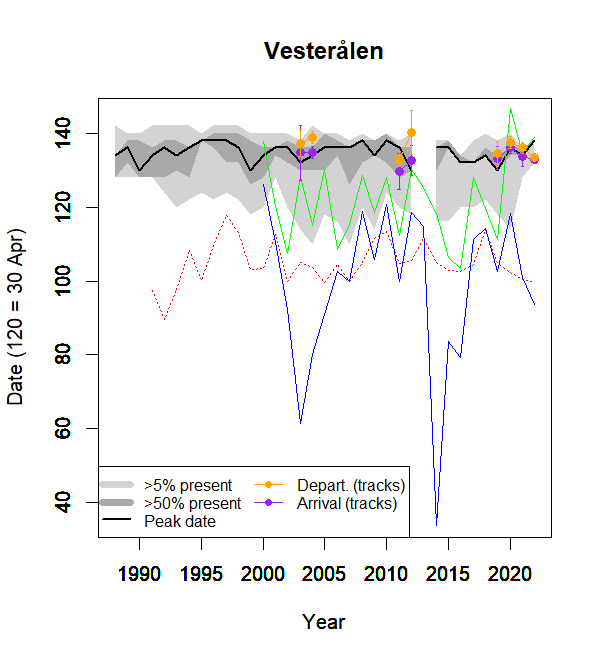

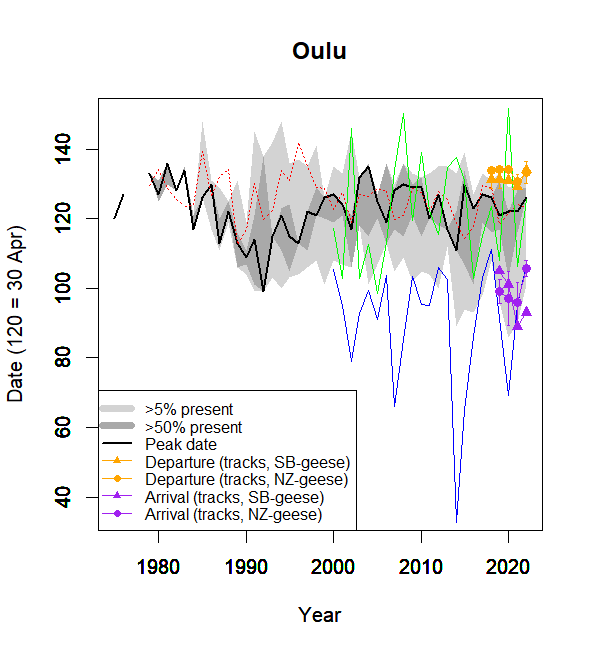

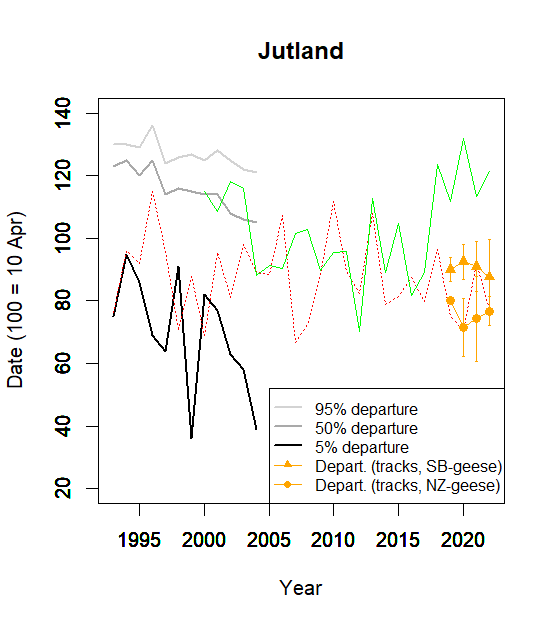

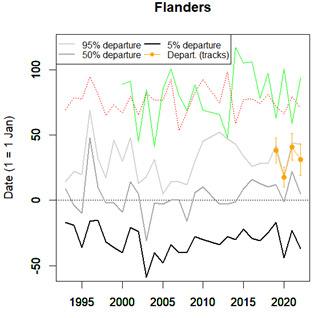


**A . Flanders**

**B . Friesland**

**C . Jutland**

**D . Örebro (New)**

**E . Trøndelag**

**F . Vesterålen**

**G . Vesterålen**

**H . Oulu (New)**

**Legend of spring measures**

**Figure S4. Egg-laying date in relation to spatial variation in spring onset.** Tracked geese nested in six different subareas in Svalbard and six subareas in Novaya Zemlya. It was tested whether differences in spring onset between subareas (within years) explained variation in egg-laying dates. Spring onset was defined based on GDD (A), snowmelt (B), and vegetation green-up (C). The relations were tested with LMMs (with random effects of year and individual) and none were significant (all p>0.08). Regressions are shown for the areas combined (dotted black line), and separated (dashed coloured lines). Red = Svalbard, blue = Novaya Zemlya.


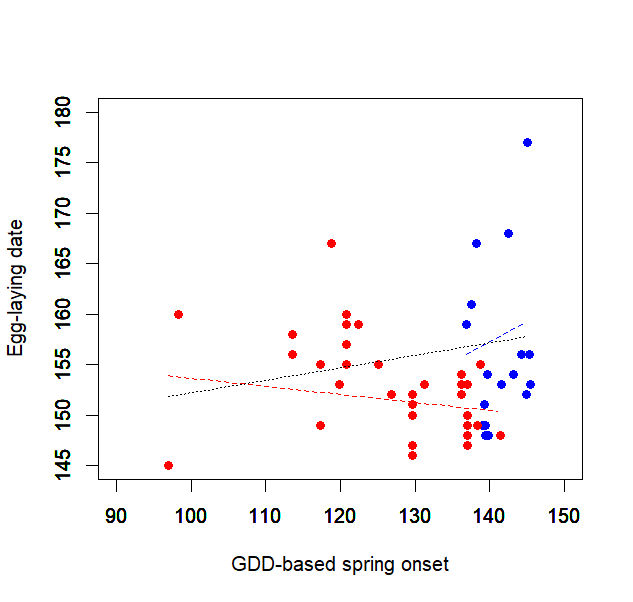

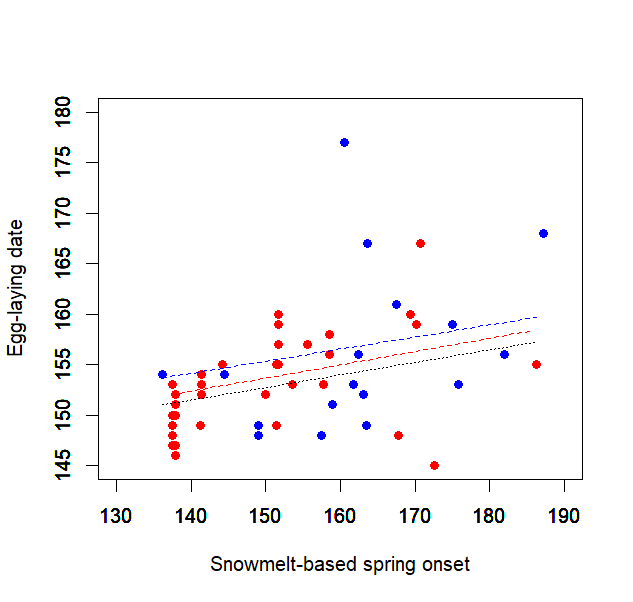

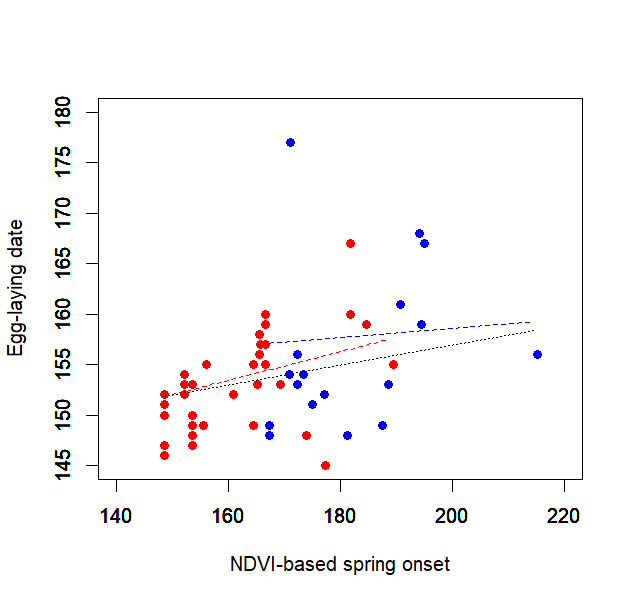


**Figure S5. Breeding propensity, success and output in traditional and newly colonised areas.** In Svalbard (traditional breeding area) and Novaya Zemlya (new breeding area), we used GPS-tracking to determine breeding propensity (chance to start breeding, panels A-B) and nesting success (chance of a nest to hatch at least one egg, panel C) of individual geese. In autumn, both subpopulations were surveyed to assess the proportion of juveniles (D). These variables are related to spring (as defined by arctic snowmelt and vegetation green-up, NDVI). In panels A-C, lines show model predictions of binomial GLMMs (on individual tracks, with random intercepts of year and individual), in panel D linear model predictions. Panel A shows regressions for both areas separate (dashed lines, but the effect of area was not significant) and the combined dataset (solid line). In panels A and B, annual means are shown for visual clarity and dot size corresponds to annual sample size. In A, C, D, data points of Svalbard and Novaya Zemlya in the same year are connected. See also Fig. 5.

D


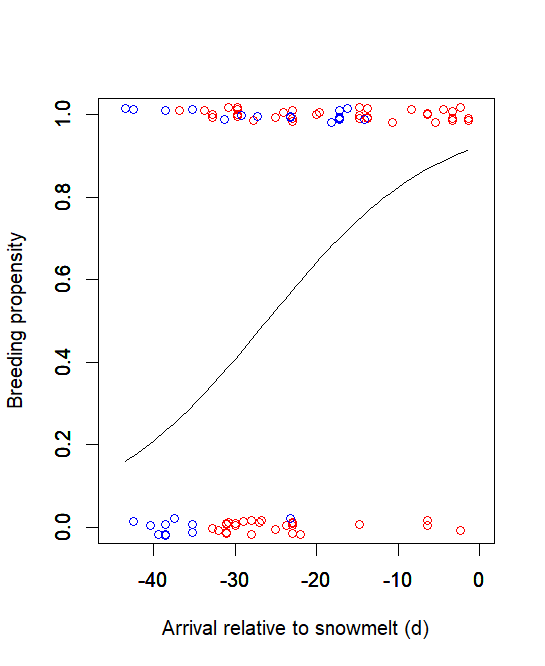

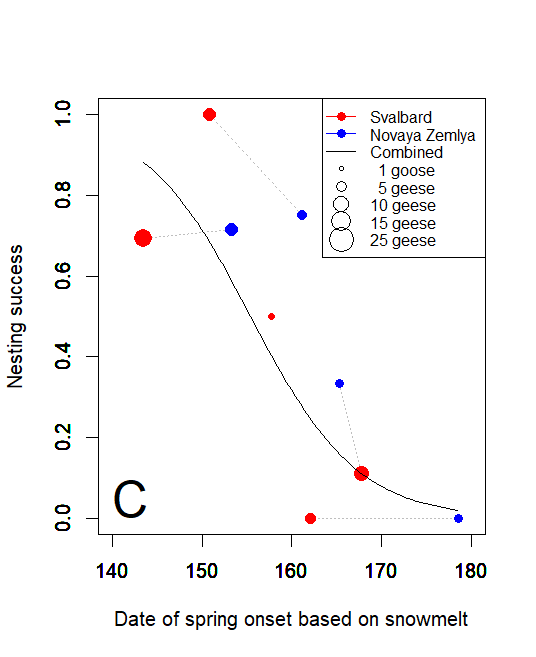

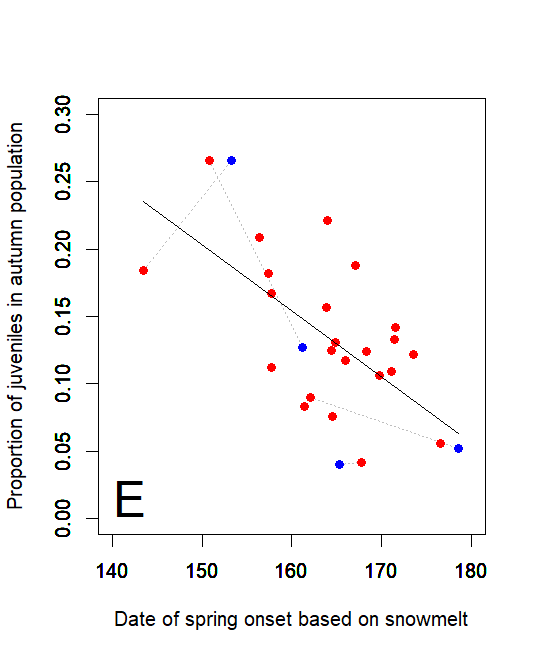


B

A

C


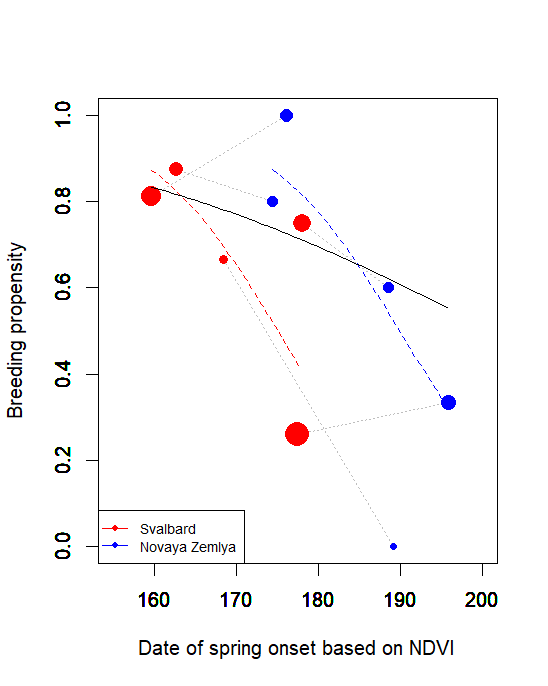


**Figure S6. Rates of climate change on the old and new migration routes, based on NDVI and snowmelt.** The onset of spring based on NDVI showed a significant advance in only two subareas of Svalbard (Sassendalen, Sauriedalen; not Svalbard average), and based on snowmelt in Svalbard overall and three of its subareas (Ekmanfjellet, Sassendalen, Sauriedalen).


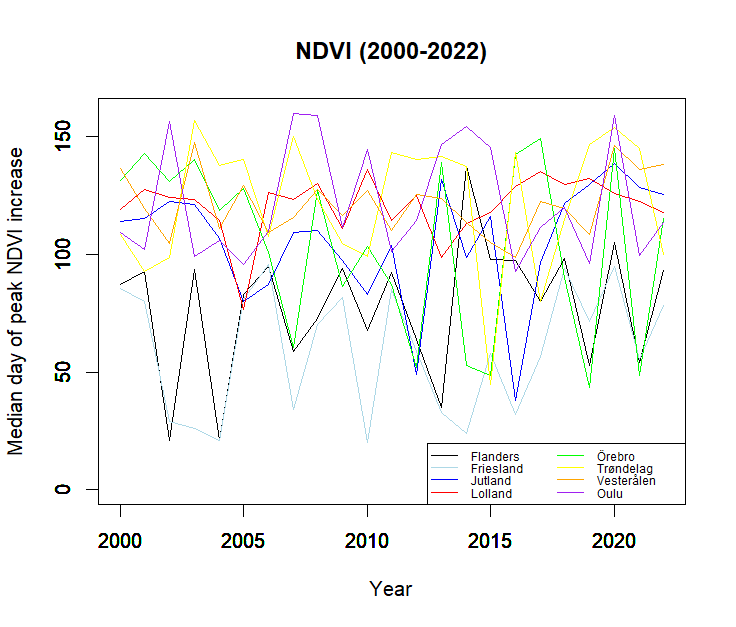

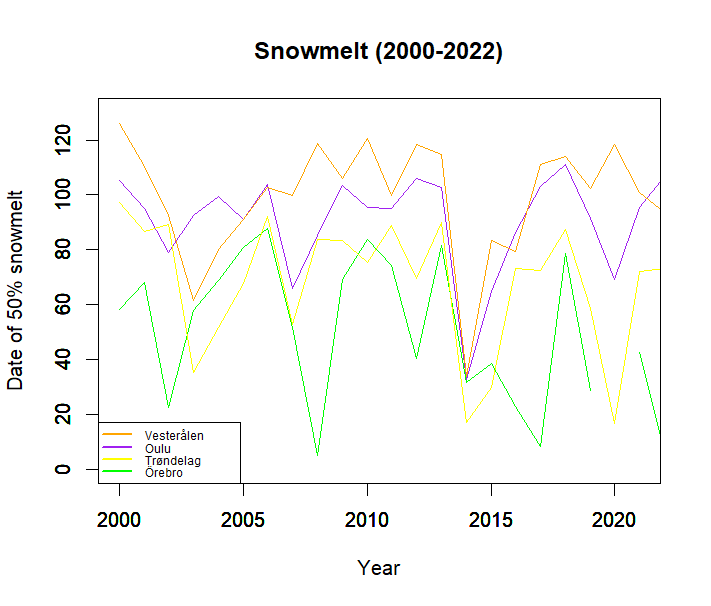


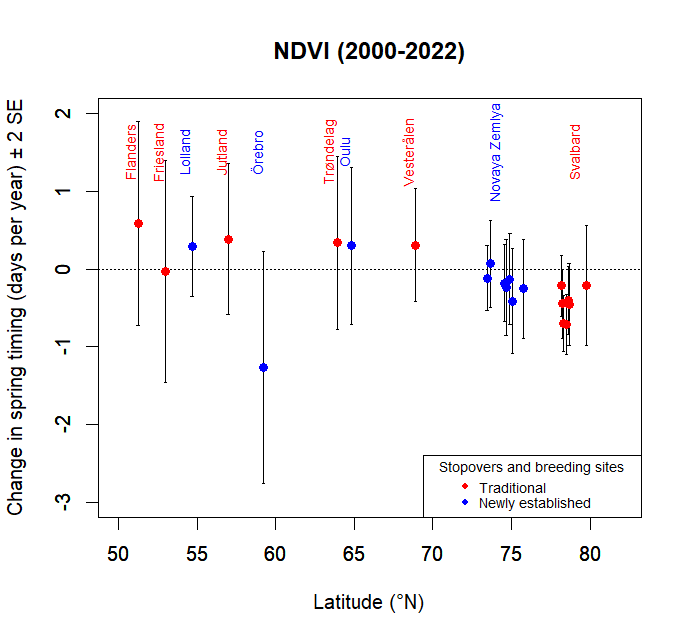

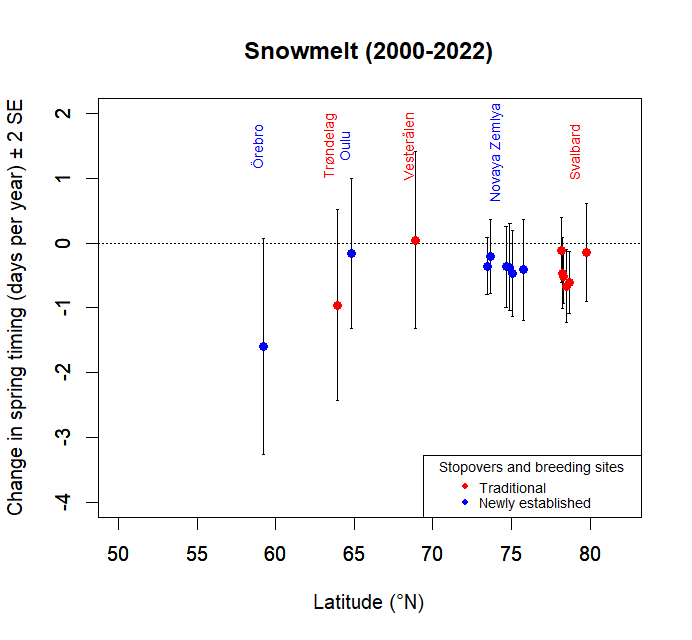


**Figure S7. Difference in spring onset between the last stopover and the breeding area.** The interval is given for different routes, from the last stopovers (Trøndelag, Vesterålen, or Oulu) to different breeding areas (Svalbard or Novaya Zemlya), based on three measures of spring onset GDD jerk, NDVI, snowmelt).


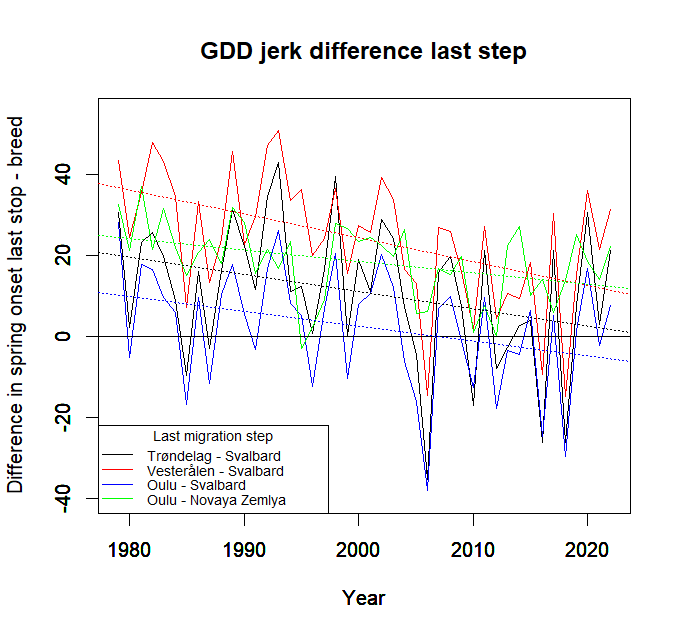

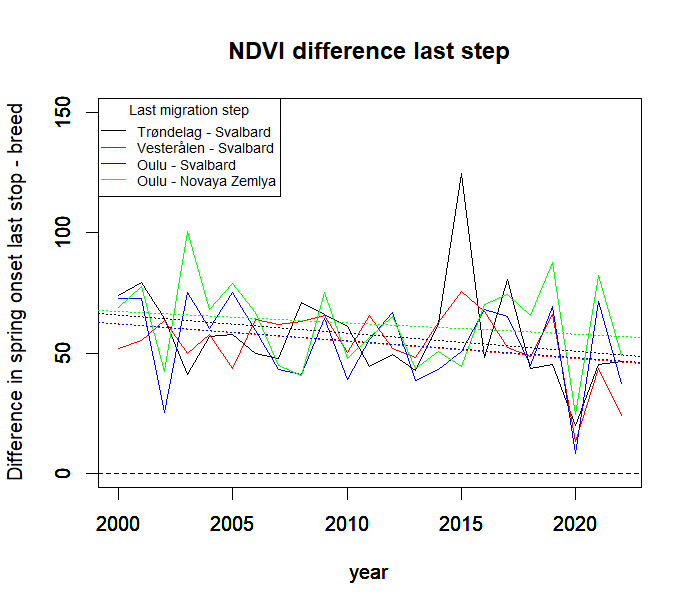


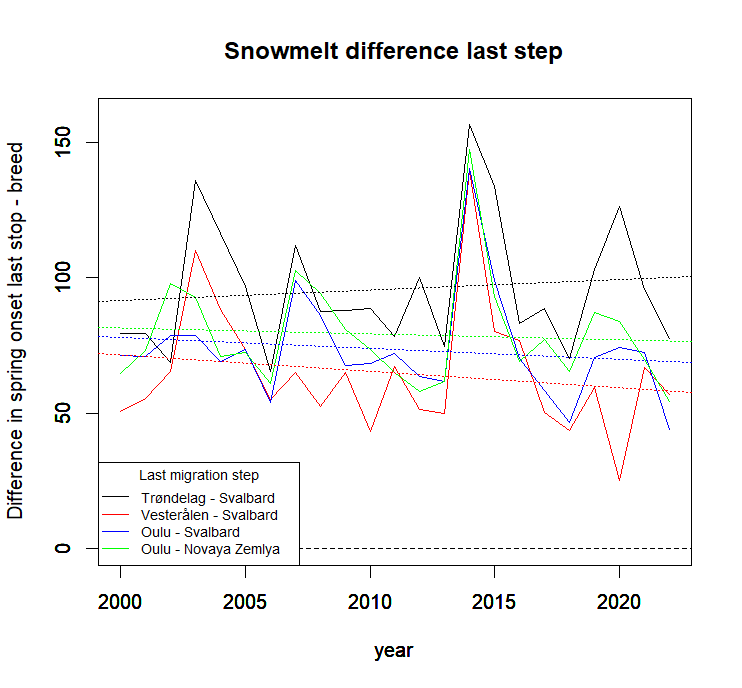

Supplement: Supplementary file 1 — Table S1. Data sources of migration at stopovers. Table S2. Sample sizes of independent tracks. Table S3. Locations of stopover sites. Table S4. Locations of subareas in Svalbard. Table S5. Locations of subareas Novaya Zemlya. Table S6. Allometry of body size measures in adult geese. Table S7. Temporal trends of GDD‐based spring over time for different sites, 1979–2022. Table S8. Predictability (spatial correlation) of spring onset for different migration steps, with spring based on GDD jerk, NDVI increase and snowmelt. Table S9. Stopover duration of individually GPS‐tracked geese (2019–2022). Table S10. Correlation between spring measures. Table S11. Correlations of migration measures in Vesterålen. Figure S1. Trends in spring onset in subareas in Svalbard and Novaya Zemlya. Figure S2. Timing of snowmelt closely around nesting sites compared with the wider area. Figure S3. The timing of spring onset and migration on different stopovers. Figure S4. Egg‐laying date in relation to spatial variation in spring onset. Figure S5. Breeding propensity, success and output in traditional and newly colonised areas. Figure S6. Rates of climate change on the old and new migration routes, based on NDVI and snowmelt. Figure S7. Difference in spring onset between the last stopover and the breeding area. [file JANE-95-97-s001.docx]
